# Somato-dendritic Synaptic Plasticity and Error-backpropagation in Active Dendrites

**DOI:** 10.1371/journal.pcbi.1004638

**Published:** 2016-02-03

**Authors:** Mathieu Schiess, Robert Urbanczik, Walter Senn

**Affiliations:** 1 Department of Physiology, University of Bern, Bern, Switzerland; 2 Center for Cognition, Learning and Memory, University of Bern, Bern, Switzerland; École Normale Supérieure, College de France, CNRS, FRANCE

## Abstract

In the last decade dendrites of cortical neurons have been shown to nonlinearly combine synaptic inputs by evoking local dendritic spikes. It has been suggested that these nonlinearities raise the computational power of a single neuron, making it comparable to a 2-layer network of point neurons. But how these nonlinearities can be incorporated into the synaptic plasticity to optimally support learning remains unclear. We present a theoretically derived synaptic plasticity rule for supervised and reinforcement learning that depends on the timing of the presynaptic, the dendritic and the postsynaptic spikes. For supervised learning, the rule can be seen as a biological version of the classical error-backpropagation algorithm applied to the dendritic case. When modulated by a delayed reward signal, the same plasticity is shown to maximize the expected reward in reinforcement learning for various coding scenarios. Our framework makes specific experimental predictions and highlights the unique advantage of active dendrites for implementing powerful synaptic plasticity rules that have access to downstream information via backpropagation of action potentials.

## Author Summary

Error-backpropagation is a successful algorithm for supervised learning in neural networks. Whether and how this technical algorithm is implemented in cortical structures, however, remains elusive. Here we show that this algorithm may be implemented within a single neuron equipped with nonlinear dendritic processing. An error expressed as mismatch between somatic firing and membrane potential may be backpropagated to the active dendritic branches where it modulates synaptic plasticity. This changes the classical view that learning in the brain is realized by rewiring simple processing units as formalized by the neural network theory. Instead, these processing units can themselves learn to implement much more complex input-output functions as previously thought. While the original algorithm only considered firing rates, the biological implementation enables learning for both a firing rate and a spike-timing code. Moreover, when modulated by a reward signal, the synaptic plasticity rule maximizes the expected reward in a reinforcement learning framework.

## Introduction

One of the fascinating and still enigmatic aspects of cortical organization is the widespread dendritic arborization of neurons. These dendrites have been shown to generate dendritic spikes [1–3] that support local dendritic processing [4–7], but the nature of this computation remains elusive. An interesting view is that the dendritic nonlinearities endow the neuron with the structure of a 2-layer neural network of point neurons, in particular if the dendrites show themselves step-like dendritic spikes, but also if the dendritic nonlinearities remain continuous [8–11]. Here we show that the dendritic morphology actually offers a substantial additional benefit over the 2-layer network. This is because it allows for the implementation of powerful learning algorithms that rely on the backpropagation of the somatic information along the dendrite that, in a network of point neurons, would not be possible in this form.

Error-backpropagation has become the classical algorithm for adapting the connection strengths in artificial neural networks [12, 13]. In this algorithm, an error at an output unit is assessed by comparing the self-generated activity with a target activity. Plasticity in hidden units is driven by this error that propagates backwards along the connections of the network. Synapses, however, transmit information just in one direction, making it difficult to implement error-backpropagation in biological neuronal circuitries. But this is different for dendritic trees. In the 2-layer structure of a dendritic tree information at the output site may be physically backpropagated across the intermediate computational layer to the synapses targeting the tree.

While the suggested dendritic error-backpropagation is a plasticity rule for supervised learning, it is also suitable for reinforcement learning. Instead of imposing the somatic spiking to learn pre-assigned target spike timings, the somatic spikes can be generated by the dendritic inputs alone, while learning is driven by a delayed reward signal. The synapse itself can be agnostic about the coding and the learning scenario; it learns by continuously adapting synaptic strength according to molecular mechanisms that are identical in the different scenarios.

Various experimental work revealed that synaptic plasticity depends on the precise timing between pre- and postsynaptic action potentials [14, 15] and the postsynaptic voltage [16]. It has further been shown that the specific form of this spike-timing-dependent plasticity (STDP) may vary with the synaptic location on the dendritic tree [17–19], and that synaptic plasticity in general is modulated by dendritic spikes [20–22]. Yet, no coherent view on the impact of dendritic nonlinearities on plasticity has emerged. Correspondingly, beside an early attempt to assign a fitness score to dendritic synapses [23] and the suggestion of a Hebbian-type plasticity rule for synapses on active dendrites [24], no computational framework for synaptic plasticity with regenerative dendritic events exists that would guide its experimental exploration. In our previous study, we derived a reward-maximizing plasticity rule that incorporates dendritic spikes, but no online implementation was presented [25]. Here, starting from biophysical properties of NMDA conductances [26], we consider an integrated somato-dendritic spiking model that captures the main biological ingredients of dendritic spikes and that is simple enough to derive an online plasticity rule for different coding schemes in the context of both supervised and reinforcement learning.

## Results

### Neuron model

**Fig 1:**
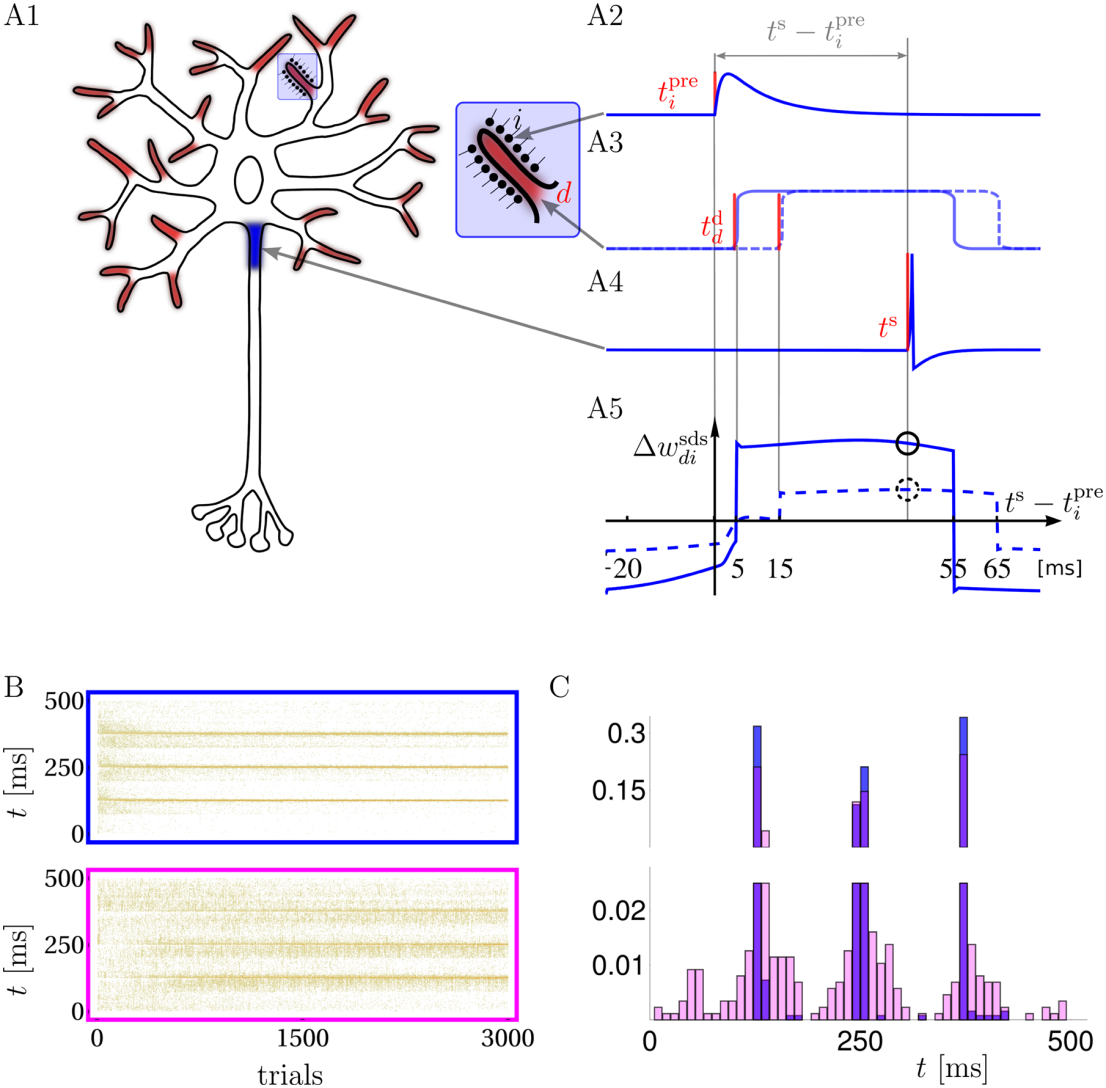
Neuron model, synaptic plasticity rule and learning of spike timings. **A**: Synaptic inputs targeting dendritic NMDA activation zones (A1, red endings with enlargement) propagate, together with possible NMDA-spikes, to the somatic spike trigger zone (A1, blue). Individual postsynaptic potentials in a dendritic branch (PSPs, arriving e.g. at time $t_{i}^{\text{pre}}$, A2), may trigger NMDA-spikes, e.g. at time $t_{d}^{d}=5\;\text{ms}$ (solid) or 15 ms (dashed) after $t_{i}^{\text{pre}}$, forming a local dendritic plateau potential of 50 ms duration (A3). A somatic spike triggered at *t*^s^ during the ongoing NMDA-spike (A4) causes a synaptic weight change $\Delta w_{di}^{\text{sds}}$ that is large/small depending on whether the NMDA-spike was triggered 5/15 ms after the presynaptic spike (A5, solid/dashed circle, respectively). A5: $\Delta w_{di}^{\text{sds}}$ as a function of $t^{s}-t_{i}^{\text{pre}}$ for a NMDA-spike at 5 (solid) and 15 ms (dashed). **B**: Raster plots of freely generated somatic spikes from test trials that are interleaved with learning trials. For the full somato-dendritic synaptic plasticity rule (sdSP) the somatic spikes converge to the 3 target times with a precision of ±3 ms (top), while the rule neglecting the dendritic spikes (i.e. suppressing the term ${\dot{w}}_{di}^{\text{sds}}$) achieves a precision of only ±14 ms (bottom). **C**: The two spike distributions from C after 3000 presentations.

We model a multi-compartment neuron with several active dendritic branches, each directly linked to a somatic compartment (Fig 1A1). The subthreshold dendritic voltage in branch *d* is the weighted sum of normalized postsynaptic potentials (PSPs) triggered by the presynaptic spikes in the afferents projecting to that branch, $u_{d}^{d}\left(t\right)=\sum_{i}w_{di}\;{\text{PSP}}_{i}\left(t\right)$. Here, *w*_*di*_ represents the synaptic strength of the synapse from afferent *i* onto branch *d* that scales the PSP amplitude. The dendritic branches can generate temporally extended NMDA-spikes of a fixed amplitude, similar to experimental observations *in vitro* [1, 3, 5] and *in vivo* [7]. In our model an NMDA-spike is represented by a square voltage pulse of amplitude *a* and duration Δ = 50 ms (Fig 1A2–1A3). It is stochastically elicited with a rate that is an increasing function of the local subthreshold membrane potential $u_{d}^{d}$ and, implicitly, of the local glutamate level. In fact, in an *in vivo* scenario the joint voltage and glutamate condition for triggering an NMDA-spike effectively reduces to a single condition on the local voltage alone. This is because the depolarization required to activate the NMDA receptors is only reached when enough glutamate was released, making the glutamate condition automatically satisfied at high enough voltages (see S1 Text).

The subthreshold dendritic voltage $u_{d}^{d}\left(t\right)$ and the dendritic spike train NMDA_*d*_(*t*) in branch *d* propagate with some attenuation factor *α* to the soma where they add up with inputs from other branches to form the somatic voltage $u^{s}=\sum_{d}\alpha (u_{d}^{d}+{\text{NMDA}}_{d})-\kappa$. This voltage is also modulated by a spike reset kernel *κ*(*t*) incorporating the transient hyperpolarisation caused by each somatic spike (Fig 1A4). For supervised learning, the somatic spikes *S*(*t*) are imposed by an external input, whereas in reinforcement learning they are stochastically triggered with an instantaneous rate *ρ*^s^(*t*) that is an increasing function of the somatic potential *u*^s^ (Online Methods).

### Learning rule

We first consider a supervised learning scenario where somatic spikes *S* are enforced by one modality (e.g. a visual stimulus) while the synaptic inputs to the dendritic branches are caused by another modality (e.g. representing an auditory stimulus [27]). The strengths of the synapses on the dendrites, *w*_*di*_, are adapted in order to reproduce the somatic spike train *S*(*t*) from just the dendritic input alone, without direct somatic drive. This can be achieved by ongoing synaptic weight changes, ${\dot{w}}_{di}$, that together maximize the likelihood of observing *S* in response to this dendritic input. According to the two types of contributions to the somatic voltage, the sub- and supra-threshold dendritic voltages, $u_{d}^{d}$ and NMDA_*d*_, the synaptic weight change can also be decomposed into a sub- and suprathreshold contribution, ${\dot{w}}_{di}={\dot{w}}_{di}^{\text{ss}}+{\dot{w}}_{di}^{\text{sds}}$, that take into account the subthreshold somato-synaptic (ss) and the suprathreshold somato-dendro-synaptic (sds) drive. We also refer to ${\dot{w}}_{di}$ as somato-dendritic synaptic plasticity (sdSP).

The somato-synaptic contribution is proportional to the postsynaptic error term (*S* − *ρ*^s^) times the local postsynaptic potential PSP_*i*_ induced by synapse *i* on that dendritic branch,

$$\begin{array}{c} {\dot{w}}_{di}^{\text{ss}}\propto \left(S-\rho^{s}\right)\cdot {\text{PSP}}_{i}\;. \end{array}$$ (1)

This corresponds to the gradient learning rule that was previously derived for a single compartment neuron [28] and that was shown to be consistent with the experimentally observed STDP (see e.g. [29]). The error term in the rule ensures that if the rate *ρ*^s^ is too small for generating *S*, the weight is increased, and if the rate is too high, the weight is decreased, eventually leading in average to 〈*S*〉 = *ρ*^s^.

The main sdSP-effect stems from the somato-dendro-synaptic contribution ${\dot{w}}_{di}^{\text{sds}}$. The instantaneous synaptic weight change at time *t* is induced by the dendritic activity Den_*d*_ in branch *d* during the interval Δ prior to *t*. Any NMDA-spike elicited in this interval will affect the somatic voltage at time *t*, and the likelihood of a dendritic spike is itself influenced by the local synaptic potentials PSP_*i*_ arriving in this interval and a few milliseconds before (Fig 1A2–1A5). Overall, we obtain an expression of the form

$$\begin{array}{c} {\dot{w}}_{di}^{\text{sds}}\propto \left(S-\rho_{\setminus d}^{s}\right)\cdot {\text{Den}}_{d}\;*\;{\text{PSP}}_{i}\;, \end{array}$$ (2)

where Den_*d*_ ∗ PSP_*i*_ captures the impact of synapse *i* on the triggering of an NMDA-spike in the preceding interval Δ, and $\rho_{\setminus d}^{s}$ represents the instantaneous somatic firing rate in the absence of a dendritic spike in branch *d* (see Online Methods). A positive error term $\left(S-\rho_{\setminus d}^{s}\right)$ tells the synapses on branch *d* how worth it is to increase their weights in order to trigger a local NMDA-spike; a negative error term suggests to rather decrease the weights since even without NMDA-spike from that branch the somatic firing rate, in average, is too high. When only dendritic nonlinearities without spiking are present, the rule Eq (2) simplifies to a pure 3-factor rule composed of a somatic difference factor, a dendritic factor, and a presynaptic factor that can be applied to dendrites showing supra- or sublinear dendritic summations (see Eq (9) in Online Methods).

The learning rule of Eq (2) can be interpreted as error-backpropagation for spiking neurons where a somatic error signal is propagated back to the dendrites that represent the nonlinear hidden units. These hidden units further modulate the error signal depending on their impact on the output unit. Classical error-backpropagation would also adapt the weights from the hidden units to the output unit. This would correspond to adapting the impact of NMDA spikes on the somatic voltage and could be modeled as dendritic branch strength plasticity [24, 30]. For conceptual clarity we discard from this extension, but the gradient calculations could also be applied to infer an optimal learning rule for these branch strengths.

### Supervised learning with active dendrites

The overall synaptic modification, *Δw*_*di*_, induced by sdSP is obtained by integrating the instantaneous changes ${\dot{w}}_{di}$ over the stimulus duration, $\Delta w_{di}=\int_{0}^{T}{\dot{w}}_{di}\left(t\right)\;dt$. Using the decomposition ${\dot{w}}_{di}={\dot{w}}_{di}^{\text{ss}}+{\dot{w}}_{di}^{\text{sds}}$ we may also write $\Delta w_{di}=\Delta w_{di}^{\text{ss}}+\Delta w_{di}^{\text{sds}}$ and have a closer look to the somato-dendro-synaptic contribution $\Delta w_{di}^{\text{sds}}$ (Fig 1A2–1A5). We fixed a presynaptic spike at time $t_{i}^{\text{pre}}=0$ and plotted $\Delta w_{di}^{\text{sds}}$ as a function of the somatic spike time *t*^s^ for the case of a NMDA-spike at *t*^*d*^ = 5 ms and 15 ms. The dendritic spike immediately after a presynaptic spike considerably extends the classical time window for causal ‘pre-post’ potentiation to a ‘pre-dend-post’ potentiation. In fact, a presynaptic spike that was taking part in triggering a NMDA-spike may indirectly contribute also to a postsynaptic spike more than 50 ms later. In turn, synaptic depression is induced in an a-causal configuration where the somatic spike comes either before the presynaptic spike or after the NMDA-spike has already decayed.

Endowed with sdSP a neuron is able to learn precise output spike-timings as shown in Fig 1B and 1C; blue) where 3 somatic spike times were imposed during the learning. The dendritic input consisted of 100 frozen presynaptic Poisson spike trains with frequency 6Hz and duration *T* = 500 ms. The dendritic tree had 20 branches, each being targeted by a random subset of the 100 afferents with a connection probability of 0.5. After repeated pattern presentations with somatic output clamped to the target spikes, the neuron learned to generate the target output from the synaptic input alone with a precision of a few milliseconds. The high spike-time precision is lost when synapses are modified only by the somato-synaptic contribution ${\dot{w}}_{di}^{\text{ss}}$ (Fig 1B and 1C; pink). Because this plasticity contribution is blind to dendritic activity, small synaptic weight changes may cause undesired appearance or disappearance of NMDA-spikes. In this case dendritic spikes arise as unpredicted knock-on effects of synaptic plasticity. Note that the somato-synaptic contribution alone, being identical to the gradient rule [28], would be able to learn the precise spiking (as would also the rules in [29, 31]) if the neuron were note endowed with the dendritic spiking mechanism and instead would only show linear summation with passive voltage propagation.

### Reward-modulated somato-dendritic plasticity

We next considered a reinforcement learning scenario where synaptic modifications are modulated by a binary feedback signal *R* = ±1 that is applied at the end of the stimulus presentation and that assesses the appropriateness of the somatic firing pattern. While this feedback is itself an external quantity, it is assumed to induce an internal signal, e.g. in the form of a neuromodulator, that globally modulates the previously induced synaptic changes. To control the balance between reward and punishment, the internal feedback modulates the past plasticity induction by a factor (*R* − *R*_∘_) with a constant reward bias *R*_∘_.

When deriving a plasticity rule that maximizes the expected reward we again obtain the same sdSP (see Eqs (1) and (2)), but now integrated across the stimulus interval and then modulated by the feedback signal,

$$\begin{array}{c} \Delta w_{di}\propto \left(R-R_{\circ }\right)\int_{0}^{T}\left({\dot{w}}_{di}^{\text{ss}}+{\dot{w}}_{di}^{\text{sds}}\right)\;dt\;, \end{array}$$ (3)

see S1C Text. We refer to this rule as reward-modulated somato-dendritic synaptic plasticity (R-sdSP). Due to the term ${\dot{w}}_{di}^{\text{sds}}$ it is effectively a 4-factor rule of the form ‘*Δw* = *Rwrd⋅som⋅dend⋅pre*’. The intuition is that the intrinsic neuronal stochasticities generate fluctuations in the somatic spiking that deviate from the prediction made by the dendritic input and cause an ‘error’ expressed in the somatic factor $\left(S-\rho_{\setminus d}^{s}\right)$ of the rule. These fluctuations will be reinforced or suppressed by the feedback signal. As before, the synaptic modification will be strengthened if a presynaptic spike contributed to a dendritic NMDA-spike that in turn affects the somatic voltage.

### Reinforcement learning with active dendrites

**Fig 2:**
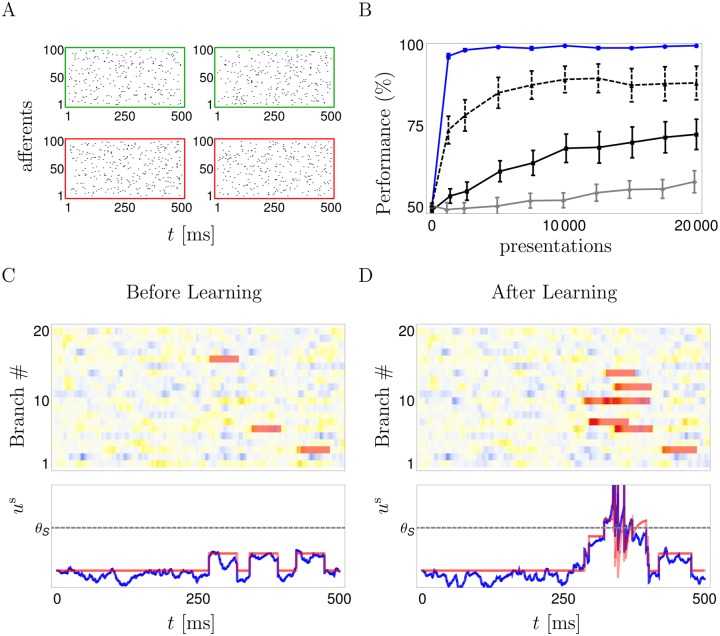
Binary classification of frozen input spike patterns by a somatic spike / no-spike code for the reward-modulated somato-dendritic synaptic plasticity (R-sdSP). **A**: Four input patterns, the two patterns in the top row should elicit no somatic spikes; the patterns in the bottom row should. **B**: R-sdSP perfectly learns the classification after roughly 1000 presentations (blue solid). In contrast, classical R-STDP fails when applied to the presynaptic–somatic (‘pre-som’, solid black) or the presynaptic–dendritic (‘pre-den’, gray) spike pairs. R-STDP improves when the dendritic spike generation is suppressed (black dashed), although it does not reach the high performance of R-sdSP. **C,D**: Dendritic and somatic voltages in response to an input pattern that requires spiking, before (**C**) and after (**D**) learning. The initially sparse dendritic spikes (NMDA_*d*_(*t*), red bars, overlaid on a $u_{d}^{d}\left(t\right)$ intensity plot) become more numerous, co-align and sum up in the soma to enable the somatic firing. Yellow indicates depolarization. Bottom: Time course of the somatic voltage *u*^s^(*t*) (blue) with the contribution of the NMDA-spikes and the somatic spike reset kernel (red).

We tested R-sdSP for various coding schemes. First, we considered a standard binary classification of frozen Poisson spike patterns by a postsynaptic spike- / no-spike code (Fig 2A). Each input pattern is defined as above (6Hz in 100 afferents for 500 ms) and belongs to one of two classes. For one class the soma is required to fire at least one spike while for the other class it should be silent. After repeated presentations followed by a reward signal, R-sdSP perfectly learned the correct classification of 4 random patterns. In contrast, reward-modulated STDP (R-STDP, [32]) implemented in its best performing version (see Online Methods and [33]) did not (Fig 2B). To achieve an appropriate alignment of dendritic spikes (Fig 2C and 2D), any successful learning rule needs to take account of the causal chain linking presynaptic spikes to dendritic and somatic spikes, the latter deciding upon reward or punishment. R-sdSP derived from maximizing the expected reward captures this causal relationship, but R-STDP does not, neither with a 10 ms (Fig 2B) nor with a 50 ms learning window (S2 Fig), and hence fails. Interestingly, R-STDP improves when the NMDA-spike generation is suppressed (Fig 2B, dashed). This shows that the increased flexibility in neuronal information processing provided by dendritic nonlinearities will in fact impede learning when a rule is used that does not take the nonlinearities into account.

R-sdSP is still able to correctly learn the classification even when the spike timings were noisy with a jitter up to 100 ms, or when the somatic voltage modulating the synaptic plasticity (via *ρ*^s^ and $\rho_{\setminus d}^{s}$) was low-pass filtered to mimic the dilution of information back-propagating to the synaptic site (S2 Fig).

Incidentally, the same task from Fig 2 can also be solved in a supervised scenario e.g. with the tempotron where, beside telling a neuron whether it should spike or not spike in response to a stimulus, the neuron is supposed to have access to the time of the voltage maximum within the stimulus interval [0, T], see e.g. [34, 35]. Although with these additional assumptions learning in principle becomes faster, the rules will again suffer from the ignorance about NMDA spikes and the possible acausality between a presynaptic spike and an immediately following somatic spike.

### Learning direction selectivity and nonlinear separation

**Fig 3:**
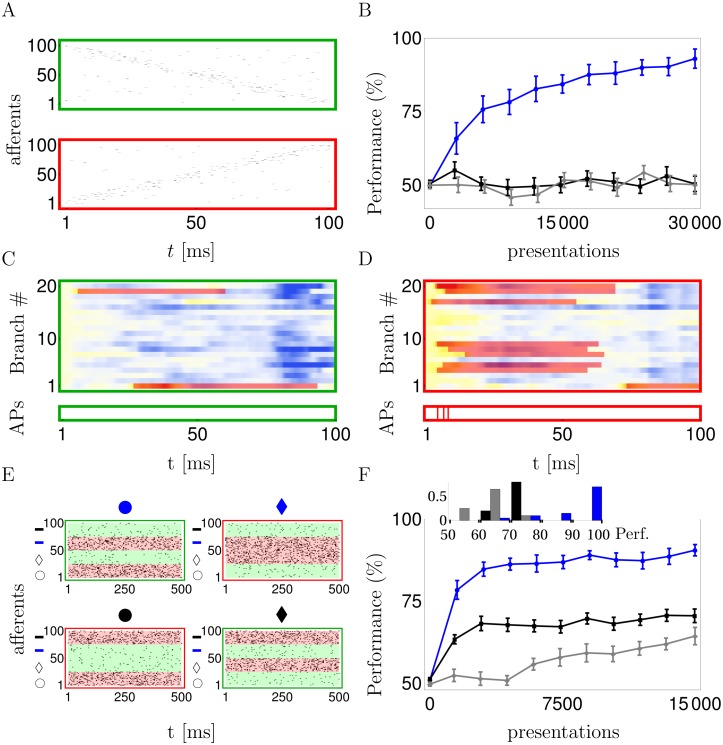
R-sdSP can exploit the representational power endowed by active dendrites. **A**: Example of presynaptic firing pattern that requires the neuron to be silent (green) or to elicit at least one somatic spike (red). **B**: R-sdSP (blue), but not R-STDP, learns to become direction selective (black: ‘pre-som’; grey: ‘pre-den’). **C, D**: The subthreshold dendritic voltages $u_{d}^{d}\left(t\right)$ and NMDA traces NMDA_*d*_(*t*) in response to the two input patterns shown in A (color code as in Fig 2). Individual branches developed direction selectivity (green). Bottom: action potentials are only generated for one direction. **E**: The 4 input patterns of the linearly non-separable feature-binding problem combine one of two shapes (‘circle’ or ‘diamond’) with one of two fill colors (‘blue’ or ‘black’). Each of the four features is represented by 25 afferents (next to the corresponding symbol on the y-axes) that encode its presence or absence by a high (40Hz) or low (5Hz) Poisson firing rate, respectively. **F**: R-sdSP learns the correct response to the 4 inputs, R-STDP does not (line code as above). Inset: average performance of each run after learning.

To apply the dendritic learning to a biological example we consider the direction selectivity of pyramidal neurons that was found to be mediated by nonlinear dendritic processing *in vitro* [5] and *in vivo* [7]. To mimick directional inputs moving in the stimulus space from right to left and left to right, we randomly enumerated the synapses across the whole dendritic tree and stochastically activated these synapses once in increasing and once in decreasing order (Fig 3A). After the stimulation, a positive reward signal was applied to the synapses when at least one somatic spike was elicited during the left-to-right patterns, or no somatic spike was elicited during a right-to-left pattern. A negative reward signal was applied in the other cases. R-sdSP, but not R-STDP, could learn such direction selectivity (Fig 3B). Individual dendritic branches may become selective to the synaptic activation order and learn to generate NMDA-spikes that, after summation in the soma, eventually trigger somatic action potentials (Fig 3C and 3D). Hence, the neuron learned to employ the dendritic nonlinearities to achieve direction selectivity, even though solving the task does not require them.

A classical task that exceeds the representational power of a point neuron is the XOR (exclusive-or) problem that is equivalent to the linearly non-separating feature binding problem [24]. In this task, the neuron has to respond exclusively to two disjoint pairs of features (e.g. to black & circle and to blue & diamond), but not to the cross combinations of these features (black & diamond and blue & circle). The presence and absence of a feature was encoded in a high and low Poisson firing rate, respectively, of a subpopulation of afferents projecting to our classifying neuron (Fig 3E). R-sdSP on the active dendrites could learn the correct responses, although due to the intrinsic stochasticity failures occurred in some cases. Classical R-STDP failed also in solving the feature binding problem problem on the dendritic tree, whether applied to *pre-dend* or to *pre-som* spike pairings (Fig 3F).

### Learning spike timings with delayed reward

**Fig 4:**
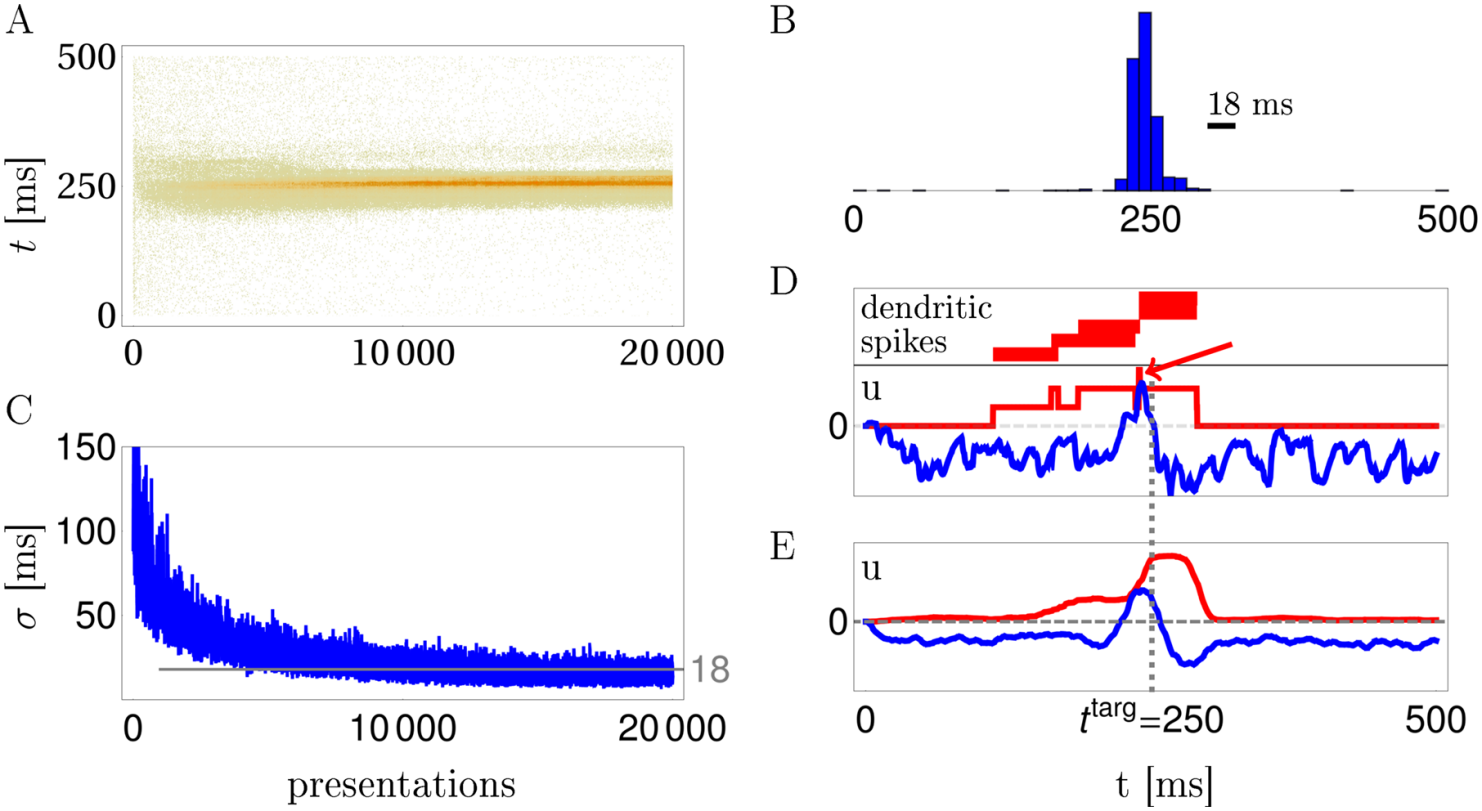
R-sdSP learns exact somatic spike timing. **A**: Somatic spike trains during 20000 trials in a reward based scenario. **B**: The distribution of somatic spikes after learning of the target time at 250 ms has a Gaussian half-width of 18 ms. **C**: Evolution of the width (*σ*) of the spike-time distribution during training. **D,E**: Separation of the somatic voltage into a contribution from the NMDA-spikes (red) and the subthreshold dendritic potentials (blue) for a single run (D) and averaged across 20 runs (E). Note that after learning the summed NMDA-spikes can form a narrow depolarizations at the target time beyond the duration of an individual spike (arrow in D).

Besides learning a spike / no-spike code or a firing rate code, R-sdSP can also learn a spike timing code, i.e. to fire only at specific times. In a first task showing this, the neuron had to learn to spike at a target time *t*^targ^ = 250 ms in response to a frozen Poisson spike pattern. Deviations from this time were punished at the stimulus ending, using a graded feedback signal that increases with the magnitude of the deviation (Online Methods). During repeated pattern presentations, while applying R-sdSP and the delayed punishing signal, the postsynaptic spiking becomes concentrated in a narrow time window around the target spike (Fig 4A–4C). To understand the role of the active dendrites we separated the time course of the somatic voltage into the contribution from the subthreshold dendritic potentials and the NMDA-spikes (Fig 4D and 4E). After successful learning, the averaged NMDA-spikes form a broad ridge around *t*^targ^ on top of which the subthreshold dendritic voltages act as ‘scorers’. Before and immediately after *t*^targ^ the subthreshold voltage is hyperpolarized to prevent somatic spikes from coming too early or too late. Notice that in an individual run the summed NMDA-spikes can form plateaus that are much shorter than the NMDA-spike duration of 50 ms. In the example shown, this arises because just 5 ms after the initiation of an NMDA-spike in one branch another NMDA-spike ends in a second branch, cutting the somatic plateau short to 5 ms (Fig 4D). By virtue of the backpropagated somatic activity, R-sdSP learns to coordinate the timing of the NMDA-spikes in the different branches, creating a narrow window for a somatic spike around the target time.

**Fig 5:**
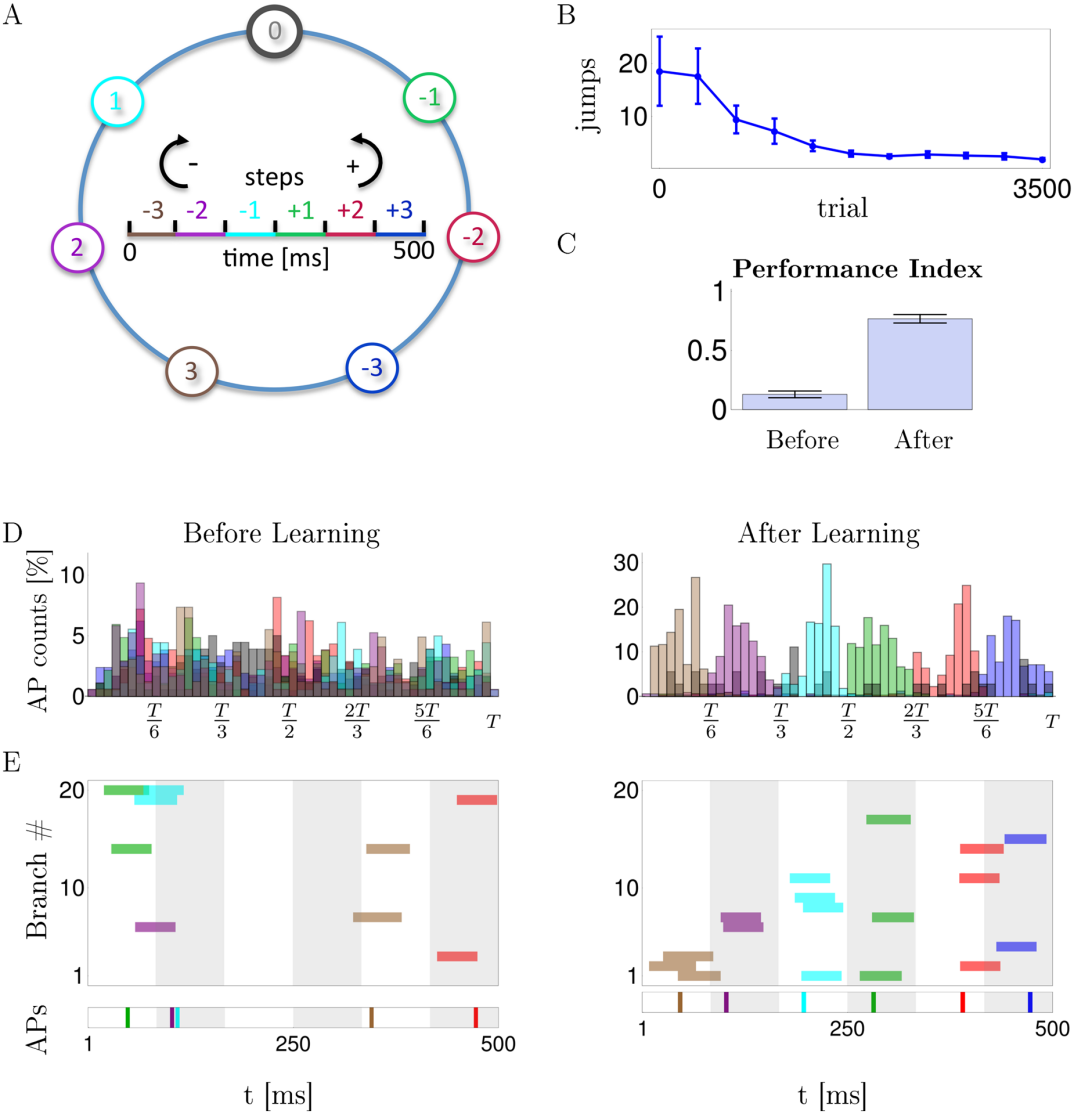
R-sdSP learns the correct spike-timing in a navigational task with binary and delayed feedback. **A**: At each position a fixed spike pattern is presented, and the timing of the first somatic spike tells how many steps in the clock (−) or counter clock (+) direction are taken. Color code of the time bin indicates the preferred spike timing for directly jumping to target position 0 when being at the correspondingly colored circle position (see text). **B**: Evolution of the mean number of jumps needed from a randomly chosen circle position until 0 is reached. **C**: Performance Index defined as the probability of directly jumping from any of the 6 circle positions to the target, and staying there if already at 0. Before learning this probability is 0.13, after learning it is 0.78. **D**: Histogram of first somatic spikes at the various positions before and after learning, averaged across patterns and learning runs (color code as in A). (**E**) Timing of the first NMDA spike in each of the 20 branches (upper panel) and the first somatic spike (lower panel) when stimulated with the patterns associated to the 6 circle positions (colors encode positions as in A). After learning, NMDA-spikes in 2-4 branches co-align and trigger somatic spike timing the appropriate time bin.

Learning a spike-timing code is also possible if the rewarding / punishing signal is binary and is potentially delayed by several stimulus durations. We conceived a spatial navigation task where 7 positions on a circle are encoded each by a frozen 500 ms spike pattern in 100 afferents projecting to the dendritic branches of the model neuron as before. The task is to jump to position 0 when being in one of the other 6 positions and, after reaching position 0, staying there (Fig 5A). Actions consisted in either no jump or jumps of 1, 2 or 3 steps clock or counter clockwise. No jump is encoded by no somatic spikes, and a jump of *n* steps in the clock or counterclockwise direction is encoded by the first somatic spike arising in the *n*’th time bin to the left or right from the center (Fig 5A, inset). A positive reward signal *R* = 1 is delivered when the agent, being in a non-target position, directly jumps to the target, or when it is at the target position and stays there; else *R* = −1. After an initial average of 20 random actions needed to reach the target position, the R-sdSP modulated agents learned to eventually reach the target with a single action and stay there (Fig 5B and 5C). While initially the first somatic spike times of our model neuron were uniformly distributed across the 500 ms stimulus interval, the neuron eventually learned to respond in the appropriate time bin of 83 ms duration that encoded the correct jumps (Fig 5D). During learning, the dendritic branches develop a shared selectivity for the patterns and until the first NMDA-spikes become properly aligned in the correct time bin (Fig 5E).

## Discussion

We derived a synaptic plasticity rule for synapses on active dendrites that minimizes errors in the supervised and maximizes reward in the reinforcement learning scenario. More precisely, the rule follows the gradient of (a lower bound of) the log-likelihood of reproducing a given spike train for supervised learning, and the gradient of the expected reward for reinforcement learning. The rule specifies the optimal timing between the presynaptic, dendritic and somatic spikes, including the time course of the postsynaptic voltages. We showed that neurons can only exploit the increased representational power of active dendrites when synaptic plasticity is modulated by both the somatic and the dendritic spiking. The suggested somato-dendritic spike-dependent synaptic plasticity (sdSP) learns to correctly respond to synaptic input patterns coding by either frozen spikes times or firing rates, while classical STDP fails. It is remarkable that the same plasticity induction that supports the learning of precise spiking in the supervised learning scenario also maximizes the expected reward when modulated by an internal and possibly delayed reward signal, irrespective of whether the postsynaptic code is based on spike times or firing rates.

The neuron model for which we derived the gradient rules considered dendritic spikes as saturating square-shaped depolarizations triggered by the crossing of a dendritic voltage threshold. We showed that the dual voltage-glutamate criterion for NMDA spikes reduces in the presence of balanced excitation and inhibition to a pure voltage criterion. This is because the glutamate condition is always satisfied when reaching the voltage threshold. This leads to a dendritic spike scenario that also includes dendritic sodium [36] or calcium spikes [37] differing in their voltage threshold, duration and amplitude. In the supervised learning scenario, the general plasticity rule we derived consists of a somatic error term that measures the difference between the actual spiking and the instantaneous spike rate, a dendritic rate- and spike-term, and a presynaptic spike term. Potentiation is triggered if the presynaptic spike is followed by a postsynaptic spike within roughly 10 ms, and this time window is stretched to roughly 50 ms if between the pre- and postsynaptic spike an additional dendritic spike occurs. Plasticity, be it potentiation or depression, can also be boosted by a mere nonlinear dendritic depolarizations without dendritic spikes, linking the rule also to computational models considering nonlinear but continuous dendritic processing [8–11, 24]. In the reinforcement learning scenario, the same plasticity rule is modulated by a global reward signal.

As learning is driven by a somatic error term, the synapses must be able to readout this error by disentangling the backpropagating spike and the somatic voltage (or at least a low-pass filtered version of it, see S2C–S2F Fig). Synapses must also read out the local dendritic spike and potential, and the synapse-specific postsynaptic potential (PSP). The PSP may be inferred from the concentration of the local glutamate released by the presynaptic bouton. The somatic and dendritic spikes may be determined from their characteristic voltage upstrokes and sustained depolarizations, and the NMDA spike can be further detected by a rapid increase in the local calcium concentration. Finally, the (subthreshold) somatic and dendritic depolarization may be distinguished by co-sensing local ion concentrations. In fact, the synaptically induced dendritic depolarization goes together with an increased local sodium concentration while the backpropagating somatic voltage does not cause such a ion influx. We assume that synapses developed a molecular machinery to extract these quantities and infer approximate estimates for the terms occuring in our plasticity rules.

Our computational framework for active dendrites contributes to the debate whether plasticity on dendritic branches should depend on the dendritic rather than the somatic spike [38], or whether it subserves synaptic clustering [23, 39] or a homeostatic adaptation [40]. In fact, when seen in the light of learning, synaptic plasticity is predicted to depend on all the postsynaptic quantities. Based on the model of dendritic NMDA receptor conductances in an *in vivo* stimulation scenario, the learning rule yields a guideline for experimental testings. For instance, it is in line with the observed synaptic depression induced by a synaptically generated dendritic spike alone ([41], but see [42]), or with the extended time window for plasticity induction involving NMDA-spikes [21]. It predicts that an NMDA-spike within roughly 50 ms after an excitatory synaptic input always enhances the synaptic modification. While the sign of the synaptic modification is determined by the presence or absence of a somatic spike following the synaptic input, an additional synaptically evoked NMDA-spike will only enhance it, never revert this sign.

Dendritic structures that have been suggested to form a 2-layer network [8] offer the additional advantage of easily backpropagating the information of the output to the synaptic sites 2 layers upstream. From a computational perspective it is interesting to note that, one the one hand, 2-layer networks represent an universal function approximator [43] while, on the other hand, networks with more than two layers are difficult to be trained [13]. For the dendritic implementation this suggests to limit the internal nonlinearities to a single layer of active dendritic branches. Because stacking dendritic nonlinearities across multiple layers would cause additional cross-talk, the restriction to a single dendritic nonlinearity may just be nature’s solution to the trade-off between achieving more representational power and paying the associated signaling costs required for efficient learning.

### Online Methods

#### Neuron parameters

The postsynaptic potentials are defined by ${\text{PSP}}_{i}\left(t\right)=\sum \epsilon \left(t-t_{i}^{\text{pre}}\right)$, where the sum extends across all presynaptic spike times $t_{i}^{\text{pre}}$ of afferent *i*. The spike reset is defined by *κ*(*t*) = ∑_*t*^s^_ *κ*_∘_(*t* − *t*^s^), where the sum extends across all somatic spike times *t*^s^. The two kernels are defined as

$$\begin{array}{c} \epsilon \left(t\right)=\frac{\Theta (t)}{\tau_{m}-\tau_{s}}\left(e^{-t/\tau_{m}}-e^{-t/\tau_{s}}\right)\;,\;\kappa_{\circ }\left(t\right)=\Theta \left(t\right)e^{-t/\tau_{m}}\;, \end{array}$$

with *τ*_m_ = 10 ms and *τ*_s_ = 1.5 ms. Here, Θ(*t*) = 1 for *t* ≥ 0 and Θ(*t*) = 0 for *t* < 0. The instantaneous rate for generating a NMDA-spike in dendrite *d* is $\rho_{d}^{d}\left(t\right)\equiv \rho^{d}\left(u_{d}^{d}\left(t\right)\right)$, and the instantaneous rate of generating a somatic spike is *ρ*^s^(*t*)≡*ρ*^s^(*u*^s^(*t*)) with

$$\begin{array}{c} \rho^{d}\left(u\right)=r_{D}/\left(1+\exp\left(-\beta_{D}\left(u-\theta_{D}\right)\right)\right)\;,\;\rho^{s}\left(u\right)=\exp\left(\beta_{s}\left(u-\theta_{s}\right)\right)\;. \end{array}$$ (4)

This model of NMDA-spike generation can be deduced from the biophysical properties of NMDA receptors in a roughly balanced input scenario where the glutamate level required to activate the NMDA receptors is always reached for those voltages that also relieve their magnesium block (see S1A Text). The choice of the saturating rate function for the NMDA generation was motivated both by stability reasons, and also because the dendritic nonlinearities are saturating [11].

The neuronal parameters are *r*_D_ = 5, *β*_D_ = *β*_s_ = 5, *θ*_s_ = 2 and *θ*_D_ = 2.4. We considered *n* = 20 branches and a dendritic attenuation factor *α* = 0.06. The probability that one out of the 100 afferents is connected to a given branch was *p* = 0.5. The amplitude of the dendritic NMDA-spike is *a* = 6, its duration Δ = 50 ms. Different NMDA-spikes in the same branch add in time but not in amplitude, yielding a dendritic plateau potential in branch *d* of the form NMDA_*d*_(*t*) = *a* if at least one NMDA-spike was triggered in the interval Δ before *t* and NMDA_*d*_(*t*) = 0 else. For simplicity we assumed the two parameters *α* and *a* to be identical for all branches, but they may vary across branches or even be treated as adaptable dendritic ‘coupling strengths’ that could be learned by analogous gradient rules as suggested by experimental work [30].

#### The learning rule

To obtain an online rule that is identical in the supervised and reinforcement scenarios up to reward modulation, we consider an additional low-pass filtering of the instantaneous synaptic changes. Plasticity is then triggered when either the stimulus ends or when reward is applied. We introduce the instantaneous synaptic eligibility for somato-synaptic and the somato-dendro-synaptic contribution, respectively, by

$$\begin{array}{ccc} e_{di}^{\text{ss}}\left(t\right) & = & (S\left(t\right)-\rho^{s}\left(t\right)){\text{PSP}}_{i}\left(t\right) \end{array}$$ (5)

$$\begin{array}{ccc} e_{di}^{\text{sds}}\left(t\right) & = & (S\left(t\right)-\rho_{\setminus d}^{s}\left(t\right)){\text{Den}}_{d}\;*\;{\text{PSP}}_{i}\left(t\right) \end{array}$$ (6)

with *S*(*t*) = ∑_*t*^s^_ *δ*(*t* − *t*^s^) representing the somatic spike train and $\rho_{\setminus d}^{s}\left(t\right)$ the instantaneous somatic escape rate without contribution of the putative NMDA-spike from branch *d* at time *t*,

$$\begin{array}{c} \rho_{\setminus d}^{s}\left(t\right)=c\;\rho^{s}(u^{s}\left(t\right)-\alpha {\text{NMDA}}_{d}\left(t\right))\;. \end{array}$$ (7)

Here, $c=\left(e^{\alpha a\beta_{s}}-1\right)/\left(\alpha a\beta_{s}\right)$. Note that a low-pass filtered version of *ρ*^s^ and $\rho_{\setminus d}^{s}$ could be extracted at the synaptic site by using the local ionic concentrations to disentangle the local, synaptically generated voltage and NMDA-spike from the backpropagated somatic voltage (see also Discussion). The factor Den_*d*_∗PSP_*i*_ expresses a modulation of the synaptic signal PSP_*i*_ by the presence or absence of an NMDA-spike in branch *d* before *t*, i.e. within the time interval *t* − Δ to *t*. If there is such a spike, the last NMDA-spike initiation time at branch *d* in the interval [*t* − Δ, *t*] is denoted by $t_{d}^{d}$. We then set

$$\begin{array}{c} {\text{Den}}_{d}*{\text{PSP}}_{i}\left(t\right)=\{\begin{array}{cc} \frac{1}{\rho_{d}^{d}\left(t_{d}^{d}\right)}\rho_{d}^{d^{\prime }}\left(t_{d}^{d}\right)\;{\text{PSP}}_{i}\left(t_{d}^{d}\right) & \text{if}\;t\;\text{within}\;\text{NMDA-spike}\;\text{triggered}\;\text{at}\;t_{d}^{d}\text{,}\\ \int_{t-\Delta }^{t}\rho_{d}^{d^{\prime }}\left(t^{\prime }\right){\text{PSP}}_{i}\left(t^{\prime }\right)dt^{\prime } & \text{else}\;\text{,} \end{array} \end{array}$$ (8)

where $\rho_{d}^{d^{\prime }}=\frac{\beta_{D}}{r_{D}}\rho_{d}^{d}\left(r_{D}-\rho_{d}^{d}\right)$ represents the derivative of $\rho_{d}^{d}\left(u\right)$ with respect to $u=u_{d}^{d}$.

The upper line in Eq (8) can be understood as a sampling version of the lower line: Let *D*_*d*_(*t*) be the sum of delta-functions centered at the triggering times of NMDA-spikes in branch *d*. In the case that the NMDA-spikes in the same dendritic branch would add up, the upper line becomes $\int_{t-\Delta }^{t}\frac{D_{d}\left(t^{\prime }\right)}{\rho_{d}^{d}\left(t^{\prime }\right)}\rho_{d}^{d^{\prime }}\left(t^{\prime }\right){\text{PSP}}_{i}\left(t^{\prime }\right)dt^{\prime }$, and this averages out to yield the lower line. Since in our case the NMDA-spike triggerings are rare, the two versions for the upper line are roughly equal. We further approximated the integral in the lower line by *ς*_*di*_ defined as low-pass filtered version of the integrand, ${\dot{\varsigma }}_{di}=-\varsigma_{di}/\bar{\Delta }+\rho_{d}^{d^{\prime }}{\text{PSP}}_{i}$ with filtering time constant $\bar{\Delta }=\Delta /2$. It is also possible to define Den_*d*_∗PSP_*i*_(*t*) by any convex combination of the two lines in Eq (8), but an equal weighting of them yielded best performances in our simulations.

It is illustrative to deduce from the above formulas the limit when the dendritic spiking disappears and merely a dendritic nonlinearity remains. Remember that in deriving these formulas we allowed the NMDA spikes to add up, and since for biological frequences NMDA spikes rarely overlap, this assumption is justified. If NMDA spikes are still allowed to add up (although not to infinity), we may formally scale the NMDA amplitude, the NMDA duration and the NMDA rate function by a factor *λ* → 0, i.e. replacing *a* → *λa*, Δ → *λ*Δ and $\rho_{d}^{d}\to \rho_{d}^{d}/\lambda^{2}$ and take the limit of *λ* converging to 0. The time course of the dendritic spikes in branch *d*, NMDA_*d*_(*t*), is then replaced by the nonlinearly summed dendritic voltage $\rho_{d}^{d}\left(t\right)=\rho_{d}^{d}\left(u_{d}^{d}\left(t\right)\right)$, and the somato-dendro-synaptic contribution for synapse *i* on branch *d* (Eqs (2) and (6), respectively) becomes the 3-factor rule

$$\begin{array}{c} e_{di}^{\text{sds}}\left(t\right)=(S\left(t\right)-\rho^{s}\left(t\right))\;\rho_{d}^{d^{\prime }}\left(t\right)\;{\text{PSP}}_{i}\left(t\right)\;. \end{array}$$ (9)

This rule could be seen as a gradient version of the pair-based rule in [24] and applies to the dendritic nonlinearities considered in [8–10]. An alternative derivation of the rule Eq (9) is to consider the deterministic somatic voltage $u^{s}=\sum_{d}\alpha (u_{d}^{d}+\rho_{d}^{d}\left(u_{d}^{d}\right))-\kappa$ and derive the learning rule as in [28] for the case of the exponential somatic spike rate *ρ*^s^(*u*^s^) defined in Eq (4). The direct gradient calculation leads to the two plasticity components in Eqs (5) and (9), respectively, corresponding to the linear and nonlinear summation of the dendritic potentials $u_{d}^{d}$.

Coming back to the case with dendritic spikes, the two instantaneous eligibilities for sdSP (Eqs (5) and (6)) are again weighted and low-pass filtered,

$$\begin{array}{c} {\dot{E}}_{di}\left(t\right)=-E_{di}\left(t\right)/\tau_{E}+\bar{a}\;e_{di}^{\text{sds}}\left(t\right)+\;e_{di}^{\text{ss}}\left(t\right)\;, \end{array}$$ (10)

with $\bar{a}=a/2$ and filtering time constant *τ*_E_ = *T*/2, *T* = 500 ms. The supervised learning rule (sdSP) is obtained by clamping the somatic output to the target spike train and updating the weights after each stimulus by the synaptic eligibility trace at that time,

$$\begin{array}{c} \Delta w_{di}\left(T\right)=\eta \;E_{di}\left(T\right)\;, \end{array}$$

with optimized learning rate *η* (see below). In reinforcement learning the synaptic weights are updated at the times *t*^Rew^ when a reward signal is applied, i.e. when a stimulus ends (*t*^Rew^ = *T* in Figs 2–5). For R-sdSP we obtain

$$\begin{array}{c} \Delta w_{di}\left(t^{\text{Rew}}\right)=\eta (R-R_{0})\;E_{di}\left(t^{\text{Rew}}\right)\;. \end{array}$$

The reward signal *R* depends on the input spike pattern and somatic spike train *S*, and *R*_0_ is a baseline set to *R*_0_ = 1 for all simulations with R-sdSP. Setting *R*_0_ to a running average of the reward would speed up learning, but for simplicity we refrained from this optimization (see, however, the implementation of R-STDP in Eq (11), where *R*_0_ must even depend on the stimulus). For a derivation of the sdSP and R-sdSP as gradient of a target function see S1C Text and [25].

#### Simulation details

For all tasks and learning rules we optimized the learning rate *η* such that the performance for the optimized value *η* = *η*_∘_ is better than for the adjacent values *η* = 1.5 *η*_∘_ and *η* = *η*_∘_/1.5. In all the simulations involving R-sdSP we used the binary reward signal *R* = ±1, except for learning the very precise spike timing in Fig 4 that required a graded reward. There, *R* = 1 − ∑_*t*^som^_ *g*(*t*^som^ − *t*^targ^) with *t*^targ^ = 250 ms, $g\left(\delta \right)=1-\exp\left(-\frac{\left|\delta \right|}{T/2}\right)$, and the sum extending across all somatic spike times *t*^som^ (setting *t*^som^ = 0 when no somatic spike was emitted).

The R-STDP plasticity rule was implemented in its best performing version found in [33]. More precisely, Frémaux et al. defined the eligibility

$$\begin{array}{c} e_{di}^{\text{STDP}}\left(t\right)=A_{+}\;S\left(t\right)\int_{-\infty }^{t}\;\;x_{i}\left(s\right)\;e^{\left(s-t\right)/\tau_{+}}ds+A_{-}\;x_{i}\left(t\right)\int_{-\infty }^{t}\;\;S\left(s\right)\;e^{\left(s-t\right)/\tau_{-}}ds \end{array}$$

with $x_{i}\left(s\right)=\sum_{t_{i}^{\text{pre}}}\delta \left(s-t_{i}^{\text{pre}}\right)$ being the presynaptic spike train in afferent *i*. They set *A*_−_ = 0, *A*_+_ = 1, and *τ*_+_ matched the synaptic time constant *τ*_s_ = 10 ms. In our case the postsynaptic signal *post*(*s*) is either the somatic spike train *S*(*t*) or the local dendritic spike train *D*_*d*_(*t*) in branch *d*. As pointed out in [33], the constant baseline reward *R*_0_ used in gradient rules must be replaced by a running mean across pattern presentations that depends on the identity of the input pattern *x*, $\tilde{R}\left(x\right)=\frac{1}{\tau_{p}}R\left(x\right)+\left(1-\frac{1}{\tau_{p}}\right)\tilde{R}\left(x\right)$. Here, the history length constant is here set to *τ*_*p*_ = 5. The weight update after each stimulus presentation is

$$\begin{array}{c} \Delta w_{di}^{\text{STDP}}\left(t^{\text{Rew}}\right)=\eta (R-\tilde{R}\left(x\right))\;E_{di}^{\text{STDP}}\left(t^{\text{Rew}}\right)\;. \end{array}$$ (11)

where $E_{di}^{\text{STDP}}$ is a low-pass version of the eligibility $e_{di}^{\text{STDP}}$ with time constant *τ*_E_ (see Eq (10)).

## Supporting Information

S1 Text Supplementary Information. **A**: From the biophysics to a stochastic NMDA-spike model. **B**: Additional analysis and simulation results. **C**: Mathematical derivations.**D**: Appendix.
(PDF)

S1 Fig For balanced synaptic inputs, the NMDA-spike probability becomes a function of the voltage alone. **A**: Top: AMPA (full line), NMDA (dashed) and GABA_*A*_ (dotted) currents, at the peak conductance level, as a function of *u* defined in Eqns S1 and S2 of the Supporting Information (*α* = *β* = 0.05; excitatory currents with positive sign). Bottom: Voltage traces *u*(*t*) for 6 different synaptic drives *g*_∘_ = 0,25,50,75,100,125nS (curves from light to dark, Eq S3), with NMDA-spikes elicited by the 2 strongest *g*_∘_. **B**: *I*(*u*) (‘I–V curves’) defined by the right-hand-side of Eq S4 for the 6 synaptic drives *g*_∘_ used in A. **C**: Zero crossings *I*(*u*) = 0 of the family of curves parametrized by *g*_∘_ and 6 of with shown in B, for different inhibitory-excitatory balancing ratios *β* = 0.05 (red), *β* = 0.10 (blue) and *β* = 0.15 (green); AMPA/NMDA ratio: *α* = 0.05. **D**: The 6 voltage traces *u*(*t*) from A plotted against the glutamate time course at the NMDA receptors, *g*_∘_ *ε*^NMDA^(*t*), overlaid on the red zero-crossing curve shown in C. **E**: Whenever the Gaussian noise (red cloud) added to the mean (*g*_∘_, *u*) on the red line (center of cloud) drops into the green area, a NMDA-spike is elicited. **F**: The probability of eliciting a NMDA-spike at a given voltage (*P*(spike|*u*), top) is almost the same for the three different balancing ratios *β* that vary by a factor of 3; it is therefore roughly proportional to the instantaneous spike rate *φ*(*u*) ∝ *P*(spike|*u*) that is a function of *u* alone. Yet, because *u* as a function of *g*_∘_ saturates (C), plotting *P*(spike|*u*) versus *g*_∘_ may still give deviating curves (bottom).
(EPS)

S2 FigRobustness of R-sdSP against noise and imperfect voltage readout.**A**, **B**: When introducing a Gaussian jitter in the spike timings of the 4 frozen 6 Hz Poisson spike patterns (A) their classification into a spike / no spike code only smoothly degrades (B). Standard deviation of spike jitter: 10 ms (blue), 20 ms (red), 50 ms (green) and 100 ms (brown). **C**: The classification is still learnable by R-sdSP when the somatic voltage *u*^s^(*t*) is low pass filtered with different time constants: 5 ms (blue), 10 ms (red), 20 ms (green) and 40 ms (brown). **D**: The performance barely changes when only considering the somato-dendro-synaptic contribution ${\dot{w}}_{di}^{\text{sds}}$ of the rule (Eq (5) in Online Methods, blue dashed). On the other hand, when learning is only based on the somo-synaptic contribution (${\dot{w}}_{di}^{\text{ss}}$, Eq (4) in Online Methods) the performance degrades (magenta). Inset: performances over the first 1000 presentations. **E**, **F**: Learning curves for R-STDP when the time constant *τ*_+_ matches the NMDA-spike duration Δ = 50 ms. **E**: Still, R-STDP cannot learn a binary classification of 4 randomized spatio-temporal spike patterns, both when applied to the presynaptic–somatic spikes (solid black; dashed: performance when the NMDA-spikes are suppressed) or the presynaptic–dendritic spikes (gray). **F**: Similarly, R-STDP is not able to learn the XOR-problem (curve legend as in E). Inset: average performance after each of the 20 runs.(EPS)
