# Supplementary material for: Somato-dendritic Synaptic Plasticity and Error-backpropagation in Active Dendrites
Source: PLoS Comput Biol. 2016 Feb 3;12(2):e1004638. doi: 10.1371/journal.pcbi.1004638 (PMC4739747; doi:10.1371/journal.pcbi.1004638)
Supplement: S1 Text — A: From the biophysics to a stochastic NMDA-spike model. B: Additional analysis and simulation results. C: Mathematical derivations.D: Appendix. (PDF) [file pcbi.1004638.s001.pdf]

Somato-dendritic synaptic plasticity and error-backpropagation in active dendrites

Supplementary Information

Mathieu Schiess[1], Robert Urbanczik[1], Walter Senn[1,2]
1 Department of Physiology, University of Bern, Bern, Switzerland
2 Center for Cognition, Learning and Memory, University of Bern

---

Contents

S1A From the biophysics to a stochastic NMDA-spike model **1**
S1A.1 Biophysical model of NMDA-spikes 2
S1A.2 Reduced model of the NMDA-spike generation 4

S1B Additional analysis and simulation results **6**
S1B.1 Robustness against noise and errors in the voltage readout 6
S1B.2 Dendritic contribution to R-sdSP and comparison with R-STDP 7
S1B.3 Additional simulation details 7

S1C Mathematical derivation of the learning rules **9**
S1C.1 Derivation of the error-minimizing supervised learning rule (sdSP) 9
S1C.2 Online version of the gradient estimate 13
S1C.3 Derivation of the gradient-based reinforcement learning rule (R-sdSP) 15

S1D Appendix **17**

S1A From the biophysics to a stochastic NMDA-spike model

This Section shows that the simplified NMDA-spike model described in the main text represents a viable approximation of the full conductance-based NMDA model in the presence of an *in vivo*-like input scenario. In this scenario the AMPA and GABA_A conductances are assumed to be roughly balanced, say with GABA/AMPA ratios varying

Email address: schiess,urbanczik,senn@pyl.unibe.ch (Mathieu Schiess[1], Robert Urbanczik[1], Walter Senn[1,2]).

between 1 to 3. This implies that the high voltages where the NMDA-receptors are unblocked from the magnesium can only be reached when there is also enough glutamate present to activate them. As a consequence, the voltage alone becomes the criterium for triggering an NMDA-spike (see Fig. S1F, top).

S1A.1 Biophysical model of NMDA-spikes

Here we describe the state-of-the art biophysical model of NMDA-spike generation [1, 2, 3]. The NMDA conductance g^{N} depends on the peak conductance of a unit NMDA receptor $\bar{g}^{\mathrm{N}}$ ($= 3.9\,$[nS], see [4]), the released glutamate, and the postsynaptic voltage u. The voltage dependence is modeled by the sigmoidal function

$$\sigma(u) = \frac{1}{1 + \exp\left(-\frac{u - V^{\mathrm{N}}_{1/2}}{V^{\mathrm{N}}_{\mathrm{spread}}}\right)}$$

with $V^{\mathrm{N}}_{1/2} = -20\,\mathrm{mV}$ and $V^{\mathrm{N}}_{\mathrm{spread}} = 12.5\,\mathrm{mV}$ [1]. The time dependence of the NMDA conductance on the glutamate released at $t = 0$, is modeled by the kernel function

$$\varepsilon^{\mathrm{N}}(t) = \Theta(t) B^{\mathrm{N}} \left(e^{-t/\tau_1^{\mathrm{N}}} - e^{-t/\tau_2^{\mathrm{N}}}\right)$$

where $\Theta(t)$ is the Heaviside step function ($= 0$ for $t < 0$ and 1 else), the constants $\tau_1^{\mathrm{N}} = 40$ms and τ_2^{N} =3ms determine the rise and fall of the kernel, and the factor $B^{\mathrm{N}} = 1.33$ that normalizes the peak amplitude of ε^{N} [5]. The NMDA conductance induced by the glutamate release becomes

$$g^{\mathrm{N}} = g_\circ\, \varepsilon^{\mathrm{N}}(t)\, \sigma(u)\,.$$

Since $g_\circ$ is proportional to the peak glutamate level and as such will also scale the AMPA currents, and since in a balanced input scenario it will further be proportional to the peak inhibitory current, we will term $g_\circ$ below as synaptic drive.

Glutamate is also assumed to activate AMPA receptors that generate a total AMPA conductance proportional to the synaptic drive, $g^{\mathrm{A}} = \alpha\, g_\circ$, with proportionality factor $\alpha = 0.05$. The AMPA kernel is given by the alpha-function

$$\varepsilon^{\mathrm{A}}(t) = \Theta(t)\, \frac{t}{\tau^{\mathrm{A}}\, e} e^{-t/\tau^{\mathrm{A}}}$$

with time constant τ^{A} =5ms. The total excitatory synaptic input current to the dendritic compartment for a given peak glutamate level then becomes the sum of the

AMPA and NMDA current,

$$I^{\mathrm{E}}_{\mathrm{syn}}(t) = g_{\circ}\,\alpha\,\varepsilon^{\mathrm{A}}(t)(E_{\mathrm{A}} - u) + g_{\circ}\varepsilon^{\mathrm{N}}(t)\sigma(u)(E_{\mathrm{N}} - u) \tag{S1}$$

where $E_{\mathrm{A}} = E_{\mathrm{N}} = 0$ represent the reversal potentials for the AMPA and NMDA receptors.

We assume that the excitatory synaptic input to some degree is balanced by inhibitory synaptic input $I^{\mathrm{I}}_{\mathrm{syn}}$. The GABAergic conductance strength g^{G} is proportional to the synaptic drive for a specific glutamate level, $g^{\mathrm{G}} = \beta g_{\circ}$, and some balancing factor $\beta = 0.05$. The GABA kernel is given by an alpha function

$$\varepsilon^{\mathrm{G}}(t) = \Theta(t)\,\frac{t}{\tau^{\mathrm{G}}\,e} e^{-t/\tau^{\mathrm{G}}}$$

with τ^{G} =5ms [6]. The inhibitory current then becomes

$$I^{\mathrm{I}}_{\mathrm{syn}}(t) = g_{\circ}\,\beta\,\varepsilon^{\mathrm{G}}(t)\,(E_{\mathrm{G}} - u)\ , \tag{S2}$$

where $E_{\mathrm{G}} = -70$ is the reversal potential of the GABA$_A$ conductance. Note that for a AMPA/NMDA ratio $\alpha = 0.05$ and GABA/NMDA ratio $\beta = 0.05$ the AMPA/GABA ration becomes 1.

Besides the synaptic input to the dendritic compartment, its membrane potential is modulated by a constant leak conductance, $\bar{g}_{\mathrm{L}}$, and an additional voltage-dependent potassium conductance resulting in the K^+ inward rectifying (KIR) current [2, 3]. The KIR voltage-dependence is modeled by a sigmoidal function that monotonically decreases with increasing voltage, with half activation at $V^{\mathrm{KIR}}_{1/2} = -70$mV and spread $V^{\mathrm{KIR}}_{\mathrm{spread}}$ =12.5 [3],

$$\kappa(u) = \frac{1}{1 + \exp\left(\frac{u - V^{\mathrm{KIR}}_{1/2}}{V^{\mathrm{KIR}}_{\mathrm{spread}}}\right)}\,.$$

Overall, the membrane potential u of the dendritic compartment is governed by the dynamics

$$C_m\dot{u} = \bar{g}_{\mathrm{L}}\,(E_{\mathrm{L}} - u) + \bar{g}_{\mathrm{KIR}}\kappa(u)\,(E_{\mathrm{K}} - u) + I^{\mathrm{E}}_{\mathrm{syn}}(t) + I^{\mathrm{I}}_{\mathrm{syn}}(t)\,, \tag{S3}$$

where $E_{\mathrm{L}} = -65$mV and $E_{\mathrm{K}} = -80$mV denote the leak and potassium reversal potentials, $\bar{g}_{\mathrm{L}} = 7$nS is the leak conductance, $\bar{g}_{\mathrm{KIR}} = 8\,\bar{g}_{\mathrm{L}}$ is the KIR peak conductance [3], and $C_m = 70$nF is the membrane capacitance (yielding a time constant of $\tau_m = C_m/\bar{g}_{\mathrm{L}} = 10\,\mathrm{ms}$).

S1A.2 Reduced model of the NMDA-spike generation

We next show how the biophysical model described above can be reduced to a model in which the generation of an NMDA-spike only depends on voltage (Fig. S1), with the glutamate dependence being negligible. To justify this simplification we note that for balanced input the NMDA-spikes are triggered at roughly the same voltage independently of the glutamate level. In fact, an NMDA-spike is triggered if the voltage is high enough to unblock the magnesium, provided enough glutamate is present. Crucially, for balanced excitation and inhibition this minimal glutamate level is always reached at the unblocking voltage, and more glutamate only marginally increases the amplitude of the NMDA-spike. This limited amplitude is due to the saturation of the driving force at high voltages.

To formalize the reasoning we insert the expressions for the excitatory (S1) and inhibitory current (S2) into the dynamics for the voltage (S3). We assume that the synaptically driven input currents are all proportional to the same synaptic drive $g_\circ$ and consider the stationary solutions of

$$\begin{aligned} C_m\dot{u} =& \bar{g}_{\mathrm{L}}\,(E_{\mathrm{L}}-u)+\bar{g}_{\mathrm{KIR}}\kappa(u)\,(E_{\mathrm{K}}-u)+g_\circ\beta(E_{\mathrm{G}}-u)+ \\ &+g_\circ\alpha(E_{\mathrm{A}}-u)+g_\circ\sigma(u)(E_{\mathrm{N}}-u)\,. \end{aligned} \tag{S4}$$

Abbreviating the right-hand-side of S4 by I this translates to $C_m\dot{u} = I$ (with a positive I leading to a depolarization). For each value of u plugged into the right-hand-side of S4 this gives a total current $I(u)$. When identifying the voltage with the symbol $V \equiv u$ we obtain the classical I–V curves for different values of synaptic drives $g_\circ$. The I–V curves for the individual, synaptically driven currents AMPA, NMDA and GABA_A currents are displayed in Fig. S1A, top. Together with the leak and KIR current the form a N-shaped the overall I–V curve (Fig. S1B) that underlies the generation of the NMDA-spikes (Fig. S1A, bottom; Eq. S3). The zero-crossings of these curves, $I(u) = 0$, give the sustained voltage u for a given drive $g_\circ$ (i.e. for which $\dot{u} = 0$). These stationary voltages as a function of $g_\circ$ form the S-shaped curves in Fig. S1C, with colors indicating different balancing ratios β of excitation and inhibition. For low and high synaptic drives there is a unique stable u, but for intermediate values of $g_\circ$ two stable solutions with an intermediate unstable solution coexist.

When plotting the voltage trajectories $u(t)$ of panel A (bottom) against the time-dependent synaptic NMDA drive, $g_\circ\,\varepsilon^{\mathrm{N}}(t)$, into the $(g_\circ, u)$ phase plane, the trajectories showing an NMDA-spike make the turn around the S-shaped steady-state curve (Fig. S1, D). For a given pair synaptic drive and stationary voltage, $(g_\circ, u)$, we may ask for the likelihood that a NMDA-spike is elicited, given some Gaussian noise $\xi_{g_\circ}$ and

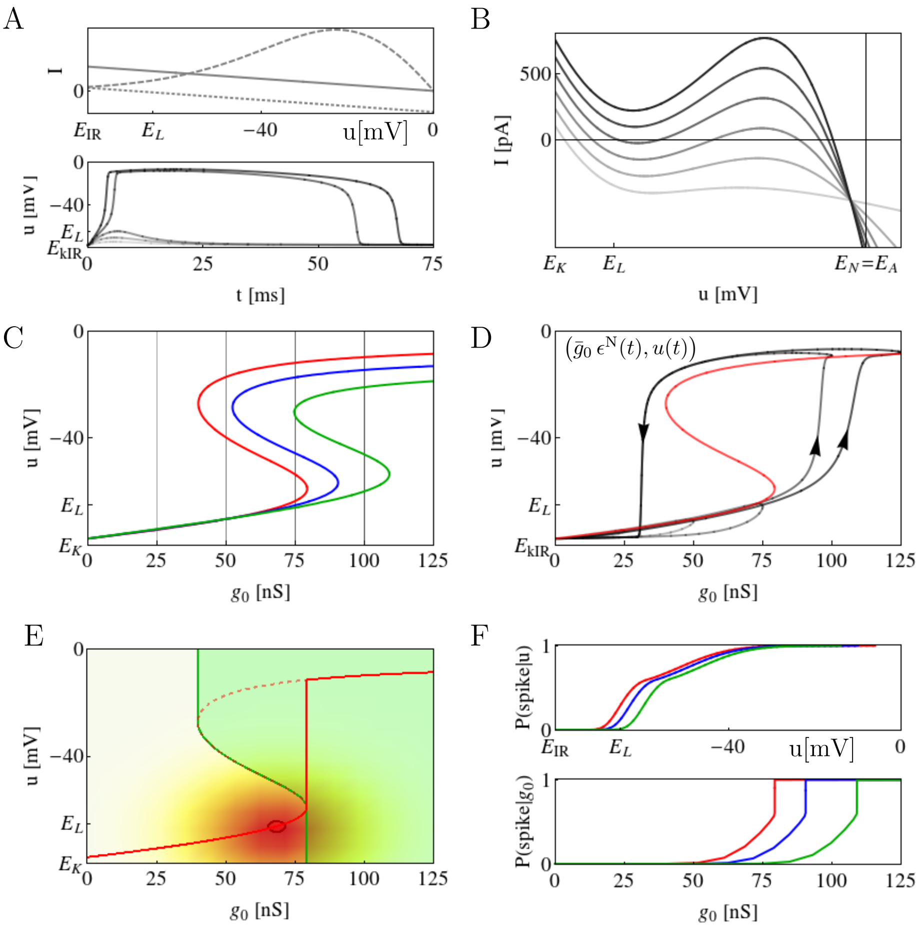

Figure S1. For balanced synaptic inputs, the NMDA-spike probability becomes a function of the voltage alone. **A**: Top: AMPA (full line), NMDA (dashed) and GABA$_A$ (dotted) currents, at the peak conductance level, as a function of u defined in Eqns S1 and S2 ($\alpha = \beta$ =0.05; excitatory currents with positive sign). Bottom: Voltage traces $u(t)$ for 6 different synaptic drives $g_\circ = 0,\ 25,\ 50,\ 75, 100, 125\,\mathrm{nS}$ (curves from light to dark, Eq. S3), with NMDA-spikes elicited by the 2 strongest $g_\circ$. **B**: $I(u)$ ('I–V curves') defined by the right-hand-side of Eq. S4 for the 6 synaptic drives $g_\circ$ used in A. **C**: Zero crossings $I(u) = 0$ of the family of curves parametrized by $g_\circ$ and 6 of with shown in B, for different inhibitory-excitatory balancing ratios $\beta = 0.05$ (red), $\beta = 0.10$ (blue) and $\beta = 0.15$ (green); AMPA/NMDA ratio: α =0.05 . **D**: The 6 voltage traces $u(t)$ from A plotted against the glutamate time course at the NMDA receptors, $g_\circ\varepsilon^{\rm N}(t)$, overlaid on the red zero-crossing curve shown in C. **E**: Whenever the Gaussian noise (red cloud) added to the mean $(g_\circ, u)$ on the red line (center of cloud) drops into the green area, a NMDA-spike is elicited. **F**: The probability of eliciting a NMDA-spike at a given voltage ($P(\text{spike}|u)$, top) is almost the same for the three different balancing ratios β that vary by a factor of 3; it is therefore roughly proportional to the instantaneous spike rate $\phi(u) \propto P(\text{spike}|u)$ that is a function of u alone. Yet, because u as a function of $g_\circ$ saturates (C), plotting $P(\text{spike}|u)$ versus $g_\circ$ may still give deviating curves (bottom).

ξ_u) added to $g_\circ$ and u, respectively (Fig. S1, E; with standard deviation $\sigma_u = 8$ and $\sigma_{g_\circ} = 3$). This likelihood is given by the probability that a point $(g_\circ + \xi_{g_\circ}, u + \xi_u)$ of the red cloud falls into the green area on the right part in panel E. When plotting the likelihood for an NMDA-spike as a function of the mean voltage that moves along the stable branch (and jumps up at the lower knee along the red line in Fig. S1E) we obtain a sigmoidal function that is roughly independent of the balancing factor β (Fig. S1F, top). Nevertheless, the same likelihood as a function of the synaptic drive (reflecting the total glutamate) yield strongly differing curves (Fig. S1F, bottom). Hence, while different balancing ratios lead to different glutamate concentrations that are required to trigger an NMDA-spike, these spikes are triggered with roughly the same likelihood at the same voltages. This justifies the stochastic NMDA-spike generation model that produces NMDA-spikes with instantaneous rate $\phi(u)$, see Fig. S1F top, as a function of the membrane potential, independently of the glutamate level.

S1B Additional analysis and simulation results

S1B.1 Robustness against noise and errors in the voltage readout

We further analyzed the robustness of the suggested reward-modulated synapto-denritic synaptic plasticity (R-sdSP) based on the classification of the 4 spatio-temporal spike patterns (as presented in Fig. 2 of the Main Text). As we have shown, the learning rule is able to classify spike patterns with frozen presynaptic spike timings and random frozen spike timings which were generated by Poisson processes with specific rates. To interpolate between these two extreme coding scenarios we also considered presynaptic spike-patterns that show stochastic spike-timings of varying degrees of stochasticity. Starting with the 4 frozen spike patterns generated once with a 6 Hz Poisson process, we perturbed each of these spike-times by a Gaussian of mean 0 and standard deviation σ (Fig. S2A, B). The learning performance shows a high robustness against these perturbations. The mean inter-spike interval for the original and perturbed spike trains are 167 ms. Even when the spike-time jitter has a width of $2\sigma = 200$ ms was the learning rule able to classify the patterns with an average performance of $\sim 90\%$ (Fig. S2B).

To explore the robustness against a dilution of the backpropagated voltage we low-pass filtered the somatic voltage $u^{\mathrm{s}}(t)$ with different time constants up to 40 ms. Learning is still possible, although it slows down with increasing filtering time constant (Fig. S2C). Note that from the low-pass filtered version $\tilde{u}^{\mathrm{s}}$ of the somatic voltage the synapse could calculate $\rho^{\mathrm{s}}_{\setminus d}(t)$ since it has access to the local NMDA-spike in branch d and hence could

subtract the contribution from the own branch. Moreover, since the passive backpropagation of the somatic voltage, the synaptic input currents and the NMDA-spikes involve different changes in ionic concentrations, a synapse sensing these concentrations may in principle disentangle the various contributions to the local voltage.

S1B.2 Dendritic contribution to R-sdSP and comparison with R-STDP

Next, we investigated the learning based on the individual components of R-sdSP. Recall that the weight change Δw_{di} of the reward gradient rule R-sdSP is composed of two components, a somato-synaptic contribution $R\,\Delta w_{di}^{\text{ss}}$ originating from the forward propagated subthreshold dendritic potential, and a somato-dendro-synaptic contribution $R\,\Delta w_{di}^{\text{sds}}$ originating from the supra-threshold dendritic plateau potentials sustained by the NMDA-spikes (Eq. 3 in the Main Text). As expected, learning based on the supra-threshold component $R\,\Delta w_{di}^{\text{sds}}$ alone is equally fast as learning based on the full R-sdSP, but the subthreshold component $R\,\Delta w_{di}^{\text{ss}}$ alone is considerably slower as it does not take account of the crucial dendritic spiking (Fig. S2D).

In the Main Text we have shown that 'classical' reward-modulated spike-timing dependent plasticity (R-STDP) [7, 8] does not reach the performance of R-sdSP (Fig. 2B and 3B,C). Here we further show that R-STDP does not perform better in the classification of the frozen spike patterns when the time constant τ_+ matches the duration of a NMDA-spike (Δ =50ms, Fig. S2E). In contrast to the gradient rule, R-STDP is not able to learn more than 75% in the presence of the dendritic spikes. The performance improves but remains below the gradient rules when the dendritic spikes are suppressed in the neuronal processing. The wider learning window in R-STDP is neither helping to improve learning for the XOR-problem that is encoded in mean firing rates (Fig. S2F).

S1B.3 Additional simulation details

In all simulations initial weights were picked independently from a Gaussian distribution with mean 0. The variance was set such that at least one somatic spike was elicited for half of the pattern presentations.

Input patterns were defined for 100 afferents. For the tasks involving temporal codes, a pattern was generated once with a constant Poisson rate of 6Hz for each afferent and the spike timings were then frozen. For the rate tasks (XOR and direction selectivity, Fig. 3 of the Main Text) each presentation was using a new realization of the pattern. For

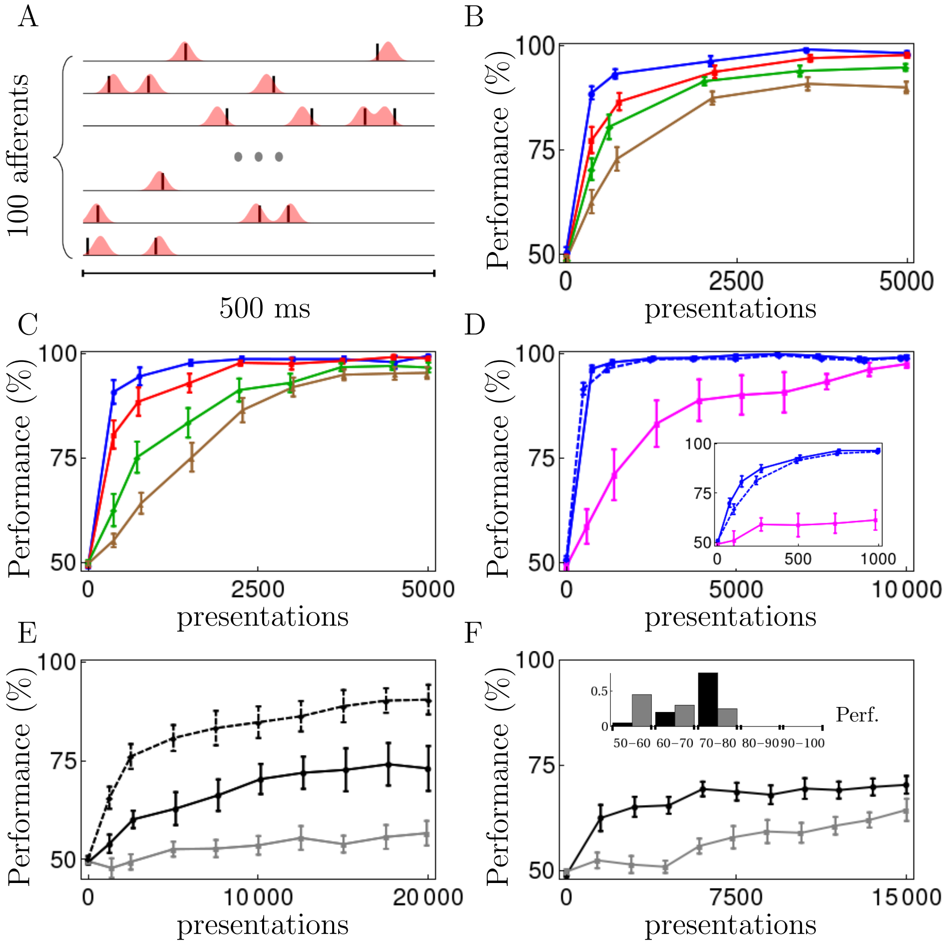

Figure S2. Robustness of R-sdSP against noise and imperfect voltage readout. **A**, **B**: When introducing a Gaussian jitter in the spike timings of the 4 frozen 6 Hz Poisson spike patterns (A) their classification into a spike / no spike code only smoothly degrades (B). Standard deviation of spike jitter: 10ms (blue), 20ms (red), 50ms (green) and 100 ms (brown). **C**: The classification is still learnable by R-sdSP when the somatic voltage $u^{\mathrm{s}}(t)$ is low pass filtered with different time constants: 5ms (blue), 10ms (red), 20ms (green) and 40 ms (brown). **D**: The performance barely changes when only considering the somato-dendro-synaptic contribution $\dot{w}_{di}^{\mathrm{sds}}$ of the rule (Eq. 5 in Online Methods, blue dashed). On the other hand, when learning is only based on the somo-synaptic contribution ($\dot{w}_{di}^{\mathrm{ss}}$, Eq. 4 in Online Methods) the performance degrades (magenta). Inset: performances over the first 1000 presentations. **E**, **F**: Learning curves for R-STDP when the time constant τ_+ matches the NMDA-spike duration Δ =50ms. **E**: Still, R-STDP cannot learn a binary classification of 4 randomized spatio-temporal spike patterns, both when applied to the presynaptic–somatic spikes (solid black; dashed: performance when the NMDA-spikes are suppressed) or the presynaptic–dendritic spikes (gray). **F**: Similarly, R-STDP is not able to learn the XOR-problem (curve legend as in E). Inset: average performance after each of the 20 runs.

the XOR problem the afferents had low (5Hz) or high (40Hz) Poisson firing rates that were again constant during the whole stimulus duration. For the direction selectivity task afferents had a low background firing rate (5Hz) replaced by a moving high firing rate interval (100Hz) of 15ms duration. An input pattern had a duration of 500ms for all tasks except the direction selectivity task which learns patterns with a duration of 100ms.

To obtain a learning curve, a running mean of the performance across presentations was computed with exponential decay constant 0.2/p, where p denotes the number of patterns to be learned. These running means were again averaged across 20 runs of the full learning for different weight and pattern initializations.

S1C Mathematical derivation of the learning rules

S1C.1 Derivation of the error-minimizing supervised learning rule (sdSP)

The aim of the supervised plasticity rule is to learn stimulus-response pairs $(\mathbf{x}, z)$ where $\mathbf{x}$ denotes a full set of presynaptic spike trains and z is the somatic spike train as a sum of delta functions ($z(t) = \sum_{t^{\mathrm{s}}} \delta(t - t^{\mathrm{s}})$, denoted as $S(t)$ in the Online Methods). Each pair $(\mathbf{x}, z)$ is drawn from a target distribution $P^*(z, \mathbf{x})$. Here we show that learning with the supervised plasticity rule maximizes a cost function. This cost function is a lower bound on the log-likelihood

$$\mathcal{L}(\mathbf{w}) = \langle \log P_{\mathbf{w}}(z|\mathbf{x}) \rangle_{P^*(z,\mathbf{x})} = \int \mathrm{d}\mathbf{x} \mathrm{d}z \, P^*(z, \mathbf{x}) \, \log P_{\mathbf{w}}(z|\mathbf{x}).$$

Note that maximizing $\mathcal{L}(\mathbf{w})$ is equivalent to minimizing the Kullback-Leibler divergence of the learned distribution P to the target distribution P^*.

In our 2-layer architecture, the conditional probability $P_{\mathbf{w}}(z|\mathbf{x})$ is not analytically tractable since the activity of dendritic branches acts as hidden variables. We denote by y_i the NMDA-spike timings of the i-th branch. In addition, the entire set of NMDA-spikes trains is denoted by $\mathbf{y} = (y_1, \ldots, y_N)$. To compute the log-likelihood, we marginalize out the hidden variables $\mathbf{y}$ in the expression,

$$\mathcal{L}(\mathbf{w}) = \left\langle \log \int \mathrm{d}\mathbf{y} \, P_{\mathbf{w}}(z|\mathbf{x}, \mathbf{y}) \, P_{\mathbf{w}}(\mathbf{y}|\mathbf{x}) \right\rangle_{P^*(z,\mathbf{x})}.$$

We apply Jensen's inequality to show that the cost function

$$\begin{aligned}\mathcal{C}(\mathbf{w}) &= \left\langle \int \mathrm{d}\mathbf{y}\, P_{\mathbf{w}}(\mathbf{y}|\mathbf{x})\, \log P_{\mathbf{w}}(z|\mathbf{x},\mathbf{y}) \right\rangle_{P^*(z,\mathbf{x})} \\ &= \langle \log P_{\mathbf{w}}(z|\mathbf{x},\mathbf{y}) \rangle_{P^*(z,\mathbf{x})\, P_{\mathbf{w}}(\mathbf{y}|\mathbf{x})} \,.\end{aligned}$$

bounds the log-likelihood from below ($\mathcal{L}(\mathbf{w}) \geqslant \mathcal{C}(\mathbf{w})$). In the sequel, the notation $\langle\cdot\rangle$ alone means that the expression is averaged over $P^*(z,\mathbf{x})\, P_{\mathbf{w}}(\mathbf{y}|\mathbf{x})$. The cost function $\mathcal{C}(\mathbf{w})$ is maximized via a stochastic gradient algorithm. The derivative of $\mathcal{C}(\mathbf{w})$ with respect to the synaptic weight w_{di} is

$$\tfrac{\partial}{\partial w_{di}} \mathcal{C}(\mathbf{w}) = \left\langle \tfrac{\partial}{\partial w_{di}} \log P_{\mathbf{w}}(z|\mathbf{x},\mathbf{y}) \right\rangle + \left\langle \log P_{\mathbf{w}}(z|\mathbf{x},\mathbf{y})\, \tfrac{\partial}{\partial w_{di}} \log P_{\mathbf{w}}(\mathbf{y}|\mathbf{x}) \right\rangle . \tag{S5}$$

As computed in [9], the gradient of the first term on the RHS is expressed as (see Eq. (4) in Online Methods)

$$\begin{aligned}\tfrac{\partial}{\partial w_{di}} \log P_{\mathbf{w}}(z|\mathbf{x},\mathbf{y}) &= \int_0^T \mathrm{d}t\, \beta_{\mathrm{s}}\, (\alpha\, \mathrm{PSP}_i(s))\, (z(t) - \rho^{\mathrm{s}}(t)) \\ &= \int_0^T \mathrm{d}t\, \beta_{\mathrm{s}}\, \alpha\, e^{\mathrm{ss}}_{di}(t).\end{aligned} \tag{S6}$$

In addition, we can manipulate the second term of the RHS to exhibit a efficient gradient estimator [10]. The procedure consists in averaging the term that accounts for the neuronal output $\log P_{\mathbf{w}}(z|\mathbf{x},\mathbf{y})$ over the hidden variable y_d at each point in time.

Let $\mathbf{y}^{\backslash d}$ denote the vector of all NMDA-spike trains but the d-th and $\mathbf{w}^{\backslash d}$ the collection of synaptic weights in all but the d-th dendritic branch. Conditioned on the input stimulus $\mathbf{x}$, each dendritic spike train is generated independently ($P_{\mathbf{w}}(\mathbf{y}|\mathbf{x}) = P_{\mathbf{w}^{\backslash d}}(\mathbf{y}^{\backslash d}|\mathbf{x})\, P_{w_{\cdot d}}(y_d|\mathbf{x})$), thus we write

$$\begin{aligned}\left\langle \log P_{\mathbf{w}}(z|\mathbf{x},\mathbf{y})\, \tfrac{\partial}{\partial w_{di}} \log P_{\mathbf{w}}(\mathbf{y}|\mathbf{x}) \right\rangle &= \int \mathrm{d}\mathbf{x}\mathrm{d}z\mathrm{d}\mathbf{y}^{\backslash d}\, P^*(z,\mathbf{x}) P_{\mathbf{w}^{\backslash d}}(\mathbf{y}^{\backslash d}|\mathbf{x})\, c'_d(w_{\cdot,d}) \\ \text{with} \quad c'_d(w_{\cdot d}) &= \int \mathrm{d}y_d\, \log P_{\mathbf{w}}(z|\mathbf{x},\mathbf{y}^{\backslash d}, y_d)\, \tfrac{\partial}{\partial w_{di}} P_{w_{\cdot d}}(y_d|\mathbf{x}) \,.\end{aligned} \tag{S7}$$

In the definition of c'_d, we can regard $\mathbf{x}$ and $\mathbf{y}^{\backslash d}$ as fixed and suppress them in the notation. To shorten the notation we use y, $w.$ and w instead of y_d, $w_{\cdot d}$ and w_{di}respectively. We now replace the Poisson process generating y ($= y_d$) by a discrete time process with step-size $\delta > 0$. The time interval $[0,T]$ is divided into K intervals of length δ. The probability to trigger a spike in interval k is

$$P_{w.}(Y_k = 1) = 1 - e^{-\delta \rho^{\mathrm{d}}(t_k)} \tag{S8}$$

where $t_k = k\,\delta$. Here the bold notation $\mathbf{Y} = (Y_1, \ldots, Y_K)$ denotes the full series of discrete binary events in the dendritic branch. With this definition, we can recover

the original Poisson process by taking the limit $\delta \to 0^+$. We denote by $\tilde{\mathbf{Y}}$ the set of NMDA-spike timings in $\mathbf{Y}$, i.e. $\tilde{\mathbf{Y}} = \{t_k | Y_k = 1\}$. Therefore, we can regard $c_d'(w)$ as the limit

$$c_d'(w) = \lim_{\delta \to 0^+} \sum_{\mathbf{Y}} \log P_{\mathbf{w}}(z|\tilde{\mathbf{Y}}) \, \tfrac{\partial}{\partial w} P_{w.}(\mathbf{Y})$$

where the sum runs over the set $\{0,1\}^K$. Since the local firing rate $\rho^{\mathrm{d}}(t_k)$ in eq. (S8) (see the Online Methods) depends only on the input $\mathbf{x}$ (y is generated by an inhomogeneous Poisson process), each Y_k are independently generated. We can express $P_{w.}(\mathbf{Y})$ as the product $P_{w.}(\mathbf{Y}^{\setminus k}) P_{w.}(Y_k)$ where $\mathbf{Y}^{\setminus k}$ denotes the full set of discrete events (spikes) but the k-th ($k = 1, \ldots, K$). Therefore, we can express the function $c_d'(w)$ as

$$c_d'(w) = \lim_{\delta \to 0^+} \sum_{k=1}^{K} \mathrm{grad}_k,$$

with

$$\mathrm{grad}_k = \sum_{\mathbf{Y}} \log P_{\mathbf{w}}(z|\tilde{\mathbf{Y}}) \, P_{w.}(\mathbf{Y}^{\setminus k}) \tfrac{\partial}{\partial w} P_{w.}(Y_k) \, .$$

We analytically compute the average of grad_k over the two outcomes $Y_k = 1$, spike at time bin k, and $Y_k = 0$, stay quiescent at time bin k. We obtain

$$\begin{aligned}
\mathrm{grad}_k &= \sum_{\mathbf{Y}^{\setminus k}} P_{w.}(\mathbf{Y}^{\setminus k}) \sum_{Y_k} \log P_{\mathbf{w}}(z|\tilde{\mathbf{Y}}) \, \tfrac{\partial}{\partial w} P_{w.}(Y_k) \\
&= \sum_{\mathbf{Y}^{\setminus k}} P_{w.}(\mathbf{Y}^{\setminus k}) \Big[\log P_{\mathbf{w}}(z|\tilde{\mathbf{Y}} \cup \{t_k\}] \tfrac{\partial}{\partial w} P_{w.}(Y_k = 1) \\
&\qquad\qquad + \log P_{\mathbf{w}}(z|\tilde{\mathbf{Y}} \setminus \{t_k\}) \tfrac{\partial}{\partial w} P_{w.}(Y_k = 0) \Big] \\
&= \sum_{\mathbf{Y}^{\setminus k}} P_{w.}(\mathbf{Y}^{\setminus k}) \Big[\log P_{\mathbf{w}}(z|\tilde{\mathbf{Y}} \cup \{t_k\}] \tfrac{\partial}{\partial w} P_{w.}(Y_k = 1) \\
&\qquad\qquad - \log P_{\mathbf{w}}(z|\tilde{\mathbf{Y}} \setminus \{t_k\}) \tfrac{\partial}{\partial w} P_{w.}(Y_k = 1) \Big] \, ,
\end{aligned}$$

where the last line follows from the identity $\frac{\partial}{\partial w} P_{w.}(Y_k = 1) = -\frac{\partial}{\partial w} P_{w.}(Y_k = 0)$. We introduce the notation

$$\gamma_{\tilde{\mathbf{Y}}}(t_k) = \log P_{\mathbf{w}}(z|\tilde{\mathbf{Y}} \cup \{t_k\}) - \log P_{\mathbf{w}}(z|\tilde{\mathbf{Y}} \setminus \{t_k\}). \tag{S9}$$

The function $\gamma_{\tilde{\mathbf{Y}}}(t_k)$ quantifies the impact that the initiation of a NMDA spike at t_k would lean on the somatic output. Since Y_k is a binary variable, the identity $\frac{\partial}{\partial w} P_{w.}(Y_k =$

$1) = (2\,Y_k - 1)\,\frac{\partial}{\partial w} P_{w.}(Y_k)$ holds independently of the value of Y_k. We deduce

$$\begin{aligned}
\mathrm{grad}_k &= \sum_{\mathbf{Y}^{\setminus k}} P_{w.}(\mathbf{Y}^{\setminus k})\gamma_{\tilde{\mathbf{Y}}}(t)\,(2Y_k - 1)\,\tfrac{\partial}{\partial w} P_{w.}(Y_k)\\
&= \sum_{\mathbf{Y}^{\setminus k}} P_{w.}(\mathbf{Y}^{\setminus k})\gamma_{\tilde{\mathbf{Y}}}(t)\,\frac{1}{2}\sum_{Y_k}(2Y_k - 1)\,\tfrac{\partial}{\partial w} P_{w.}(Y_k)\\
&= \sum_{\mathbf{Y}} P_{w.}(\mathbf{Y})\frac{\gamma_{\tilde{\mathbf{Y}}}(t)}{2}(2Y_k - 1)\,\tfrac{\partial}{\partial w}\log P_{w.}(Y_k),
\end{aligned}$$

and

$$\sum_{k=1}^{K}\mathrm{grad}_k = \sum_{\mathbf{Y}} P_{w.}(\mathbf{Y})\sum_{k=1}^{K}\frac{\gamma_{\tilde{\mathbf{Y}}}(t)}{2}(2Y_k - 1)\,\tfrac{\partial}{\partial w}\log P_{w.}(Y_k).$$

From the definition (S8), we have

$$\begin{aligned}
\tfrac{\partial}{\partial w}\log P_{w.}(Y_k = 1) &= \frac{\frac{\mathrm{d}}{\mathrm{d}u^{\mathrm{d}}}\rho^{\mathrm{d}}(t_k)}{\rho^{\mathrm{d}}(t_k)}\mathrm{PSP}(t_k) + \mathcal{O}(\delta)\\
\tfrac{\partial}{\partial w}\log P_{w.}(Y_k = 0) &= -\delta\,\tfrac{\mathrm{d}}{\mathrm{d}u^{\mathrm{d}}}\rho^{\mathrm{d}}(t_k)\mathrm{PSP}(t_k).
\end{aligned}$$

So, taking the limit $\delta \to 0^+$ and the original notation (the calculation is for the i-th synapse located in the d-th dendritic branch, see Eq. S7), we obtain

$$c_d'(w,z) = \int \mathrm{d}y_d\,P_{w\cdot d}(y_d|\mathbf{x})\int_0^T \mathrm{d}t\,\underbrace{\left[\tfrac{1}{2}\gamma_{y_d}(t)\tfrac{\mathrm{d}}{\mathrm{d}u_d^{\mathrm{d}}}\log\rho_d^{\mathrm{d}}(t)\left(y_d(t) + \rho_d^{\mathrm{d}}(t)\right)\,\mathrm{PSP}_i(t)\right]}_{e_{di}^{\mathrm{SL}}(t):=}, \tag{S10}$$

where $y_d(t)$ denotes the δ-function representation of the set y_d, $y_d(t) = \sum_{s\in y_d}\delta(t-s)$. We obtain a gradient estimate where hidden variables are partially averaged. In particular, the second term of the RHS in the equation (S5) is (see Eq. S7)

$$\left\langle \log P_{\mathbf{w}}(z|\mathbf{x},\mathbf{y})\,\tfrac{\partial}{\partial w_{di}}\log P_{\mathbf{w}}(\mathbf{y}|\mathbf{x})\right\rangle = \left\langle \int_0^T \mathrm{d}t\,e_{di}^{\mathrm{SL}}(t)\right\rangle$$

and it follows that

$$\tfrac{\partial}{\partial w_{di}}\mathcal{C}(\mathbf{w}) = \left\langle \int_0^T \mathrm{d}t\,\left(\beta_{\mathrm{s}}\,\alpha\,e_{di}^{\mathrm{ss}}(t) + e_{di}^{\mathrm{SL}}(t)\right)\right\rangle. \tag{S11}$$

This term in the brackets is our unbiased gradient estimate for the cost function $\mathcal{C}(\mathbf{w})$.

S1C.2 Online version of the gradient estimate

Here we show how an approximated version of the gradient estimate (S11) can be computed online. The central idea is to rewrite the exact gradient estimate (Eq. (S11)) with integrals that could then be implemented by low-pass filtered version. We therefore replace the rectangular integration window in the Eq. S11 by an exponential one. First, we introduce the inactivation function $\Psi_{y_d}(t)$ that depends on the dendritic spike timings y_d and that is 0 during an ongoing NMDA-spike and 1 elsewhere. Note that $\Psi_{y_d}(t)$ is related to the NMDA-spikes response function $\text{NMDA}_d(t)$ via $\text{NMDA}_d(t) = a\,(1-\Psi_{y_d}(t))$. As computed in [10], the function $\gamma_{y_d}(t)$ (Eq. S9) is given by

$$\gamma_{y_d}(t) = a\,\alpha\,\beta_\mathrm{s} \int\limits_t^{\min(T,t+\Delta)} \mathrm{d}s\,\Psi_{y_d^{\setminus t}}(s)\left(z(s)-\rho^\mathrm{s}_{\setminus d}(s)\right)$$

where $y_d^{\setminus t}$ is the set y_d with no spike timing at t ($y_d^{\setminus t} = \{s \in y_d | s \neq t\}$). In its current form, it is impossible to compute $e^\mathrm{SL}_{di}(t)$ (Eq. S10) online, since the integration of $\gamma_{y_d}(t)$ extends from the current time t into the future up to $t+\Delta$. We therefore permute the integration order to turn the integration into the future to an integration across the past (see Appendix),

$$\begin{aligned}\int_0^T dt\, e^\mathrm{SL}_{di}(t) &= \int\limits_0^T \mathrm{d}t\, \tfrac{\mathrm{d}}{\mathrm{d}u^\mathrm{d}_d} \log \rho^\mathrm{d}_d(t)\, \left(y_d(t)+\rho^\mathrm{d}_d(t)\right)\, \mathrm{PSP}_i(t) \int\limits_t^{\min(T,t+\Delta)} \mathrm{d}s\, \Psi_{y_d^{\setminus t}}(s)\, f_d(s) \\ &= \int\limits_0^T \mathrm{d}s\, f_d(s) \underbrace{\int\limits_{\max(0,s-\Delta)}^{s} \mathrm{d}t\, \Psi_{y_d^{\setminus t}}(s) \tfrac{\mathrm{d}}{\mathrm{d}u^\mathrm{d}_d} \log \rho^\mathrm{d}_d(t)\, \left(y_d(t)+\rho^\mathrm{d}_d(t)\right)\, \mathrm{PSP}_i(t)}_{\xi^\mathrm{N}_{di}(s):=}\,,\end{aligned} \tag{S12}$$

with

$$f_d(t) = \tfrac{a\,\alpha\,\beta_\mathrm{s}}{2}\left(z(s)-\rho^\mathrm{s}_{\setminus d}(t)\right).$$

Here the stimulus started at 0, therefore the synaptic signal $\text{PSP}_i(t)$ vanishes for $t<0$ and we can set $s-\Delta$ instead of $\max(0,s-\Delta)$ as a lower bound for the second integral. Our aim is to encode each integral by a low pass filter (see below). Since the function $\Psi_{y_d^{\setminus t}}(s)$ depends on the variables t and s, the function $\xi^\mathrm{N}_{di}(s)$ is generally not computable by an online procedure. In the sequel, we will see that we can drop the dependence

with respect to t in the function $\Psi_{y_d^{\setminus t}}(s)$. We start to decompose $\xi_{di}^{\mathrm{N}}(s)$ as follows:

$$\begin{aligned}\xi_{di}^{\mathrm{N}}(s) = &\int_{s-\Delta}^{s} \mathrm{d}t\, \Psi_{y_d^{\setminus t}}(s) \tfrac{\mathrm{d}}{\mathrm{d}u_d^{\mathrm{d}}} \rho_d^{\mathrm{d}}(t)\, \mathrm{PSP}_i(t) \\ &+ \int_{s-\Delta}^{s} \mathrm{d}t\, \Psi_{y_d^{\setminus t}}(s)\, y_d(t) \tfrac{\mathrm{d}}{\mathrm{d}u_d^{\mathrm{d}}} \log \rho_d^{\mathrm{d}}(t)\, \mathrm{PSP}_i(t)\,. \end{aligned} \tag{S13}$$

For a given s, the functions $\Psi_{y_d^{\setminus t}}(s)$ and $\Psi_{y_d}(s)$ are equal on the set $[s-\Delta, s]\setminus y_d$. Since y_d is a set of zero measure, we can replace $\Psi_{y_d^{\setminus t}}(s)$ by $\Psi_{y_d}(s)$ in the first integral in Eq. S13. The inactivation function $\Psi_{y_d}(s)$ vanishes if there is an ongoing NMDA spike at time s and so does the first integral in Eq. S13. Otherwise we have $\Psi_{y_d}(s) = 1$ which implies that the second integral in Eq. S13 vanishes since this integral runs only over the different NMDA-spike timings in the interval $[s-\Delta, s]$. We introduced the function $y_d(t)$ as the δ-function representation constructed from the set of individual spike times y_d and hence, if the inactivation function is 1, no NMDA-spike was initiated in $[s-\Delta, s]$. These two observations allow us to rewrite $\xi_{di}^{\mathrm{N}}(s)$ as

$$\xi_{di}^{\mathrm{N}}(s) = \begin{cases} \int_{s-\Delta}^{s} \Psi_{y_d^{\setminus t}}(s)\, \frac{\mathrm{d}}{\mathrm{d}u_d^{\mathrm{d}}} \log \rho_d^{\mathrm{d}}(t)\, y_d(t)\, \mathrm{PSP}_i(t) dt & \text{if s within a NMDA-spike,} \\ \int_{s-\Delta}^{s} \frac{\mathrm{d}}{\mathrm{d}u_d^{\mathrm{d}}} \rho_d^{\mathrm{d}}(t)\, \mathrm{PSP}_i(t) dt & \text{else}\,. \end{cases} \tag{S14}$$

In our model, spikes are triggered by point processes, a point event is called a spike timing. When two NMDA-spikes are triggered in a short interval they do not add up in amplitude but instead the second one extends the duration of the first one (see Online Methods). This renders the evaluation of $\Psi_{y_d^{\setminus t}}(s)$ complicated. In order to simplify the calculation, we assume that NMDA-spike timings are sparse. More precisely, we assume that each rectangular NMDA spike is triggered by a unique point event. Therefore, if we assume the presence of a NMDA-spike at time s which was initiated at t_d^{d} then the top integral in (S14) is $\frac{\mathrm{d}}{\mathrm{d}u_d^{\mathrm{d}}} \log \rho_d^{\mathrm{d}}(t_d^{\mathrm{d}})\, \mathrm{PSP}_i(t_d^{\mathrm{d}})$ since the inactivation function $\Psi_{y_d^{\setminus t_d^{\mathrm{d}}}}(s)$ is 1 when the unique point event which causes the current dendritic spike is removed. To summarize, we showed that

$$\xi_{di}^{\mathrm{N}}(s) = \begin{cases} \frac{\mathrm{d}}{\mathrm{d}u_d^{\mathrm{d}}} \log \rho_d^{\mathrm{d}}(t_d^{\mathrm{d}})\, \mathrm{PSP}_i(t_d^{\mathrm{d}}) & \text{if s within a NMDA-spike triggered at } t_d^{\mathrm{d}}, \\ \int_{s-\Delta}^{s} \frac{\mathrm{d}}{\mathrm{d}u_d^{\mathrm{d}}} \rho_d^{\mathrm{d}}(t)\, \mathrm{PSP}_i(t) dt & \text{else}\,. \end{cases} \tag{S15}$$

Note that in the Online Methods we put $\text{Den}_d * \text{PSP}_i(s) = \xi^{\text{N}}_{di}(s)$. Therefore, Eq. (S11) becomes (see Eq. (S12))

$$\begin{aligned}
\tfrac{\partial}{\partial w_{di}}\mathcal{C}(\mathbf{w}) &= \left\langle \int_0^T \mathrm{d}t \, \left[\tfrac{a\,\alpha\,\beta_\text{s}}{2} \left(z(s) - \rho^{\text{s}}_{\setminus d}(t) \right) \text{Den}_d * \text{PSP}_i(t) + \beta_\text{s}\,\alpha\, e^{\text{ss}}_{di}(t) \right] \right\rangle \\
&= \left\langle \alpha\,\beta_\text{s} \int_0^T \mathrm{d}t \, \left[\tfrac{a}{2} e^{\text{sds}}_{di}(t)(t) + e^{\text{ss}}_{di}(t) \right] \right\rangle .
\end{aligned}$$

In a stochastic ascent algorithm, the term in brackets defines the synaptic update

$$\Delta w_{di} = \eta \int_0^T \mathrm{d}t \, \left[\tfrac{a}{2} e^{\text{sds}}_{di}(t)(t) + e^{\text{ss}}_{di}(t) \right],$$

where η denotes the learning rate. We could eliminate the constants α and β_s in the plasticity rule since as a multiplicative constant it can be absorbed by the learning rate η. The previous update is roughly equivalent to the sdSP plasticity rule in the Main Text since the low-pass filter $E_{di}(t)$ (Eq. 8 in Online Methods) at time t represents the integration of $\tfrac{a}{2} e^{\text{sds}}_{di}(t) + \alpha\, e^{\text{ss}}_{di}(t)$ from the past to t with an exponential window (the time constant is τ_E).

S1C.3 Derivation of the gradient-based reinforcement learning rule (R-sdSP)

Here we show that the rule R-sdSP approximates an online estimate of the gradient of the expected reward

$$\bar{R}(\mathbf{w}) = \int \mathrm{d}\mathbf{x}\mathrm{d}z \, P_\mathbf{w}(z, \mathbf{x}) \, R(z, \mathbf{x})$$

We maximize $\bar{R}$ through a stochastic gradient ascent algorithm. We start to marginalize out $\mathbf{y}$

$$\begin{aligned}
\bar{R}(\mathbf{w}) &= \int \mathrm{d}\mathbf{x}\mathrm{d}\mathbf{y}\mathrm{d}z \, P_\mathbf{w}(z, \mathbf{x}, \mathbf{y}) \, R(z, \mathbf{x}) \\
&= \int \mathrm{d}\mathbf{x}\mathrm{d}\mathbf{y}\mathrm{d}z \, P_\mathbf{w}(z|\mathbf{x}, \mathbf{y}) \, P_\mathbf{w}(\mathbf{y}|\mathbf{x}) \, P(\mathbf{x}) \, R(z, \mathbf{x}) .
\end{aligned}$$

The derivative of $\bar{R}$ with respect to the synaptic weight w_{di} is

$$\tfrac{\partial}{\partial w_{di}} \bar{R}(\mathbf{w}) = \left\langle R(z, \mathbf{x}) \tfrac{\partial}{\partial w_{di}} \log P_\mathbf{w}(z|\mathbf{x}, \mathbf{y}) \right\rangle_{P_\mathbf{w}(z,\mathbf{x},\mathbf{y})} + \left\langle R(z, \mathbf{x}) \tfrac{\partial}{\partial w_{di}} \log P_\mathbf{w}(\mathbf{y}|\mathbf{x}) \right\rangle_{P_\mathbf{w}(z,\mathbf{x},\mathbf{y})} . \tag{S16}$$

The first term on the RHS of (S16) is computed in Eq. (S6) and represents the classical reward maximizing rule [9]. As previously introduced [10], we consider an alternative

gradient estimator based on the following identity

$$\left\langle R(z,\mathbf{x})\frac{\partial}{\partial w_{di}}\log P_{\mathbf{w}}(\mathbf{y}|\mathbf{x})\right\rangle_{P_{\mathbf{w}}(z,\mathbf{x},\mathbf{y})} = \left\langle R(z,\mathbf{x}) \int_0^T \mathrm{d}t\, e^{\mathrm{R}}_{di}(t)\right\rangle_{P_{\mathbf{w}}(z,\mathbf{x},\mathbf{y})}$$

where

$$e^{\mathrm{R}}_{di}(t) = \tanh\left(\tfrac{1}{2}\gamma_{y_d}(t)\right) \tfrac{\mathrm{d}}{\mathrm{d}u^{\mathrm{d}}_d}\log\rho^{\mathrm{d}}_d(t)\, \left(y_d(t)+\rho^{\mathrm{d}}_d(t)\right)\, \mathrm{PSP}_i(t).$$

More precisely, we have shown in [10] that $e^{\mathrm{R}}_{di}(t)$ is an appropriate gradient estimator for the second term on the RHS of (S16). Thus, the gradient of the expected reward can be written as

$$\frac{\partial}{\partial w_{di}}\bar{R} = \left\langle R(z,\mathbf{x}) \int_0^T \mathrm{d}t\, \left(\alpha\,\beta_{\mathrm{s}}\, e^{\mathrm{ss}}_{di}(t) + e^{\mathrm{R}}_{di}(t)\right)\right\rangle_{P_{\mathbf{w}}(z,\mathbf{x},\mathbf{y})},$$

We observe that if we perform a linear approximation of the hyperbolic tangent then we obtain $e^{\mathrm{R}}_{di}(t) \approx e^{\mathrm{SL}}_{di}(t)$. As a result, we can apply the calculation of the previous section (see *Online version of the gradient estimate*), it leads to

$$\begin{aligned}\frac{\partial}{\partial w_{di}}\bar{R} &\approx \left\langle R(z,\mathbf{x}) \int_0^T \mathrm{d}t\, \left(e^{\mathrm{SL}}_{di}(t) + \alpha\,\beta_{\mathrm{s}}\, e^{\mathrm{ss}}_{di}(t)\right)\right\rangle_{P_{\mathbf{w}}(z,\mathbf{x},\mathbf{y})} \\ &= \left\langle \alpha\,\beta_{\mathrm{s}} R(z,\mathbf{x}) \int_0^T \mathrm{d}t\, \left(\tfrac{a}{2} e^{\mathrm{sds}}_{di}(t)(t) + e^{\mathrm{ss}}_{di}(t)\right)\right\rangle_{P_{\mathbf{w}}(z,\mathbf{x},\mathbf{y})}.\end{aligned} \tag{S17}$$

and the update rule

$$\Delta w_{di} = \eta\, (R - R_0) \int_0^T \mathrm{d}t\, \left[\tfrac{a}{2} e^{\mathrm{sds}}_{di}(t)(t) + e^{\mathrm{ss}}_{di}(t)\right],$$

where η is the learning rate and R_0 is a baseline. The constant R_0 can be introduced since the update is roughly a gradient rule [11] and so the following identity holds

$$\begin{aligned}\left\langle R_0\,\alpha\,\beta_{\mathrm{s}} \int_0^T \mathrm{d}t\, \left(\tfrac{a}{2} e^{\mathrm{sds}}_{di}(t)(t) + e^{\mathrm{ss}}_{di}(t)\right)\right\rangle_{P_{\mathbf{w}}(z,\mathbf{x},\mathbf{y})} &\approx R_0 \left\langle \int_0^T \mathrm{d}t\, \left(e^{\mathrm{R}}_{di}(t) + \alpha\,\beta_{\mathrm{s}}\, e^{\mathrm{ss}}_{di}(t)\right)\right\rangle_{P_{\mathbf{w}}(z,\mathbf{x},\mathbf{y})} \\ &= R_0 \int \mathrm{d}\mathbf{x}\mathrm{d}\mathbf{y}\mathrm{d}z\, \frac{\partial}{\partial w_{di}} P_{\mathbf{w}}(z,\mathbf{x},\mathbf{y}) \\ &= R_0 \frac{\partial}{\partial w_{di}} \underbrace{\int \mathrm{d}\mathbf{x}\mathrm{d}\mathbf{y}\mathrm{d}z\, P_{\mathbf{w}}(z,\mathbf{x},\mathbf{y})}_{=1} \\ &= 0.\end{aligned}$$

where the approximation sign accounts for the linear approximation of tanh in the definition of $e^{\mathrm{R}}_{di}(t)$. As observed at the end of the previous section, the integral of the

update rule is roughly equal to the value of the low pass-filter $E_{di}(t)$ (Eq. 8 in Online Methods) at time T, yielding the plasticity rule cited in the Main Text.

Overall, we made two approximations: (1) The NMDA spikes were supposed to be sparse in time such that they do not overlap and we could neglect the voltage saturation. With an NMDA spike duration of 50 ms the approximation error is small if the NMDA spike rate is smaller than 20 Hz. (2) The symmetric tanh has been linearized around 0. Importantly, both approximations never change the sign of any component of the true gradient vector. Hence, although after the approximations the learning rule may deviate from the true gradient, it will still be hill climbing. Note that both target functions, the lower bound of the log-likelihood for supervised learning and the expected reward for reinforcement learning, are everywhere continuous (in fact differentiable, but in general not convex), so that learning with these approximations still smoothly maximizes these target functions (locally).

S1D Appendix

Here we detail the steps from the first to the second line in the formula (S12).

We rewrite the equation (S12) in a form that allows the permutation of the integration order,

$$\int_0^T \mathrm{d}t\, e_{di}^{\mathrm{SL}}(t) = \int_0^T \mathrm{d}t\, \zeta_{di}(t) \int_0^T \mathrm{d}s\, \chi_{[t,t+\Delta]}(s)\, \Psi_{y_d^{\setminus t}}(s)\, f_d(s),$$

with

$$\zeta_{di}(t) = \tfrac{\mathrm{d}}{\mathrm{d}u_d^{\mathrm{d}}} \rho_d^{\mathrm{d}}(t)\, \left(y_d(t) + \rho_d^{\mathrm{d}}(t)\right)\, \mathrm{PSP}_i(t),$$

and where $\chi_{[t,t+\Delta]}(s)$ denotes the indicator function

$$\chi_{[t,t+\Delta]}(s) = \begin{cases} 1 & \text{if } s \in [t, t+\Delta] \\ 0 & \text{else} \end{cases}.$$

Now we change the integration order

$$e_{di}^{\mathrm{Caus}}(z, \mathbf{y}, \mathbf{x}) = \int_0^T \mathrm{d}s\, f_d(s) \int_0^T \mathrm{d}t\, \chi_{[t,t+\Delta]}(s)\, \Psi_{y_d^{\setminus t}}(s)\, \zeta_{di}(t).$$

The inequalities $s \leqslant t \leqslant s+\Delta$ is equivalent to $t-\Delta \leqslant s \leqslant t$, therefore the two functions $\chi_{[t,t+\Delta]}(s)$ and $\chi_{[s-\Delta,s]}(t)$ too. As initially, the action of $\chi_{[s-\Delta,s]}(t)$ is equivalent to change

the boundaries of the second integral, we deduce

$$\int_0^T \mathrm{d}t\, e_{di}^{\mathrm{SL}}(t) = \int_0^T \mathrm{d}s\, f_d(s) \int_0^T \mathrm{d}t\, \chi_{[s-\Delta,s]}(t)\, \Psi_{y_d^{\setminus t}}(s)\, \zeta_{di}(t)$$
$$= \int_0^T \mathrm{d}s\, f_d(s) \int_{\max(0,s-\Delta)}^{s} \mathrm{d}t\ \Psi_{y_d^{\setminus t}}(s)\, \zeta_{di}(t).$$

References

[1] G. Major, A. Polsky, W. Denk, J. Schiller, and D. W. Tank. Spatiotemporally graded NMDA spike/plateau potentials in basal dendrites of neocortical pyramidal neurons. *J. Neurophysiol.*, 99:2584–2601, May 2008.

[2] H. Sanders, M. Berends, G. Major, M. S. Goldman, and J. E. Lisman. NMDA and GABAB (KIR) conductances: the "perfect couple" for bistability. *J. Neurosci.*, 33(2):424–429, Jan 2013.

[3] G. Major, M. E. Larkum, and J. Schiller. Active properties of neocortical pyramidal neuron dendrites. *Annu. Rev. Neurosci.*, 36:1–24, Jul 2013.

[4] B. F. Behabadi and B. W. Mel. Mechanisms underlying subunit independence in pyramidal neuron dendrites. *Proc. Natl. Acad. Sci. U.S.A.*, 111(1):498–503, Jan 2014.

[5] F. Gabbiani, J. Midtgaard, and T. Knopfel. Synaptic integration in a model of cerebellar granule cells. *J. Neurophysiol.*, 72(2):999–1009, Aug 1994.

[6] P. Rhodes. The properties and implications of NMDA spikes in neocortical pyramidal cells. *J. Neurosci.*, 26(25):6704–6715, Jun 2006.

[7] E. Izhikevich. Solving the distal reward problem through linkage of STDP and dopamine signaling. *Cerebral Cortex*, 17:2443–2452, 2007.

[8] N. Frémaux, H. Sprekeler, and W. Gerstner. Functional requirements for reward-modulated spike-timing-dependent plasticity. *J. Neurosci.*, 30:13326–13337, Oct 2010.

[9] J. Pfister, T. Toyoizumi, D. Barber, and W. Gerstner. Optimal spike-timing-dependent plasticity for precise action potential firing in supervised learning. *Neural Computation*, 18:1318–1348, 2006.

[10] M. E. Schiess, R. Urbanczik, and W. Senn. Gradient estimation in dendritic reinforcement learning. *The Journal of Mathematical Neuroscience*, 2(2), 2012.

[11] R. Williams. Simple statistical gradient-following algorithms for connectionist reinforcement learning. *Machine Learning*, 8:229–256, 1992.
